# Long‐term multi‐species demographic studies reveal divergent negative impacts of winter storms on seabird survival

**DOI:** 10.1111/1365-2656.14227

**Published:** 2024-11-19

**Authors:** Kirsty Laurenson, Matt J. Wood, Tim R. Birkhead, Matthew D. K. Priestley, Richard B. Sherley, Annette L. Fayet, Tim Guilford, Ben J. Hatchwell, Stephen C. Votier

**Affiliations:** ^1^ Lyell Centre Heriot Watt University Edinburgh UK; ^2^ University of Gloucestershire Cheltenham UK; ^3^ School of Biosciences University of Sheffield Sheffield UK; ^4^ Department of Mathematics & Statistics University of Exeter Exeter UK; ^5^ Environment and Sustainability Institute and Centre for Ecology and Conservation University of Exeter Penryn UK; ^6^ Norwegian Institute for Nature Research Trondheim Norway; ^7^ Department of Zoology University of Oxford Oxford UK

**Keywords:** climate change, guillemot, mark‐recapture, non‐breeding, puffin, razorbill

## Abstract

1. Understanding storm impacts on marine vertebrate demography requires detailed meteorological data in tandem with long‐term population monitoring. Yet most studies use storm proxies such as the North Atlantic Oscillation Index (NAOI), potentially obfuscating a mechanistic understanding of current and future risk.
2. Here, we investigate the impact of extratropical cyclones by extracting north Atlantic winter storm characteristics (storm number, intensity, clustering and wave conditions) and relating these with long‐term overwinter adult survival of three long‐lived sympatric seabirds which winter at sea—common guillemot *Uria aalge*, Atlantic puffin *Fratercula arctica* and razorbill *Alca torda*.
3. We used multidecadal mark‐recapture analysis (1970s–2020s) to estimate survival while correcting for resighting probability, combined with spatially explicit environmental data from geolocation‐derived wintering areas, to determine the impact of different storm characteristics (i.e., number, intensity, duration, gap between storms, wave height and wind speed), as well as broad‐scale climatic conditions (NAOI and sea surface temperature [SST]).
4. All three species experienced rapid population growth over the study period. Guillemot and razorbill survival was lower during stormier winters, with an additive effect of summer SST for guillemots, and a negative interaction with population size for razorbills. Puffin survival was negatively correlated with winter SST, and the lowest puffin survival coincided with intense winter storms and a large seabird wreck in 2013/14. The number of days with wind speed >30 and 35 ms^−1^ negatively impacted razorbill and guillemot survival, respectively, and puffin survival was higher when gaps between storms were longer.
5. Our results suggest negative but divergent storm impacts on these closely related sympatric breeders, which may be compounded by warmer seas and density‐dependence as these populations return to their previously much larger sizes. We tentatively suggest that frequent, long‐lasting storms with strong winds are likely to have the greatest negative impact on auk survival. Moreover, we highlight the possibility of tipping points, where only the most extreme storms, that may become more frequent in the future, have measurable impacts on seabird survival, and no effect of NAOI.

## Graphical abstract

We combine multidecadal mark‐recapture data with storm and environmental data from wintering areas derived by tracking to compare storm impacts on survival of three seabird species frequently associated with wrecks (common guillemot *Uria aalge*, Atlantic puffins *Fratercula arctica* and razorbills *Alca torda*), and investigate whether storm characteristics can signal wrecks.

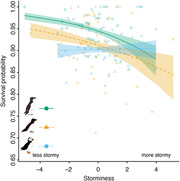

## 1 INTRODUCTION

Increasing global sea and air temperatures are having profound ecological consequences (Parmesan & Yohe, 2003; Sydeman et al., 2015), but the impact of increasingly severe and variable climate extremes is especially worrying (Diffenbaugh et al., 2017). This is exemplified in marine ecosystems where anthropogenic forcing has altered storm patterns, increasing the frequency and intensity of both tropical and extratropical storms (Kossin et al., 2020; Priestley et al., 2020). As the climate continues to warm, extreme storms are expected to become more frequent (Priestley & Catto, 2022), making it crucial to understand their ecological impact.

Many marine vertebrates are at risk from increasing storms, but their slow life histories (e.g. delayed maturation and long life spans) mean their populations change slowly over time, making it challenging to quantify demographic effects (Schreiber & Burger, 2002). Indeed, this strategy of low and variable annual fecundity in favour of high and stable adult survival with multiple lifetime reproductive attempts may have evolved partly in response to environmental unpredictability (Dobson & Jouventin, 2007). Such life history strategies therefore require long‐term longitudinal studies to determine the potential impact of changing storms. Moreover, assessing storm impacts for migratory marine vertebrates may be further complicated because these animals can disperse over wide oceanic areas, making it difficult to link environmental impacts at the appropriate spatio‐temporal scale (Costa et al., 2012).

Climate change, including more frequent storms, is considered one of the top three threats to seabirds in terms of number of species affected and average impact (Dias et al., 2019). During breeding, storm waves may flood nests and reduce breeding success (Newell et al., 2015) while during non‐breeding, which forms the largest portion of the year and coincides with peak storm frequency at high latitudes, storms may starve seabirds by reducing foraging efficiency or prey availability (Clairbaux et al., 2021). However, the demographic cost of more frequent and intense winter storms is still not fully understood. Low winter survival in adult seabirds has been linked to large‐scale seasonal climate indices such as the North Atlantic Oscillation Index (NAOI), El Niño Southern Oscillation (ENSO) and Southern Ocean Index (SOI) (e.g. Boano et al., 2010; Genovart et al., 2013; Votier et al., 2005). While these covary with some aspects of storms (Hurrell, 1995), they may poorly represent localised weather conditions relevant to monitored populations (Walz et al., 2018). Moreover, they are unable to tease apart the characteristics of storms such as wind, waves and clustering (Priestley et al., 2017), limiting our ability to determine the mechanism of impacts. For instance, rough sea conditions limit diving seabirds' foraging ability (Birkhead, 1976; Clairbaux et al., 2021), but it is unclear whether wind strength, sea conditions, storm duration, storm frequency or a combination of these factors are the most important drivers of ecological impacts. Other factors such as competition, food availability and pollution may also be important as additive or synergistic sources of mortality (Votier et al., 2005). Given the above, examining the effect of storm characteristics on seabird mortality is important to anticipate species‐specific risk and recovery strategies (e.g. Sydeman et al., 2021).

Accurately estimating winter storm effects on seabird fitness correlates requires long‐term capture‐mark‐recapture (CMR) studies combined with detailed weather observations (Guery et al., 2019; Reiertsen et al., 2021). Here, we examined the multidecadal (1970s–2020s) impact of winter storms on the survival of sympatrically breeding common guillemots (*Uria aalge*), razorbills (*Alca torda*) and Atlantic puffins (*Fratercula arctica*) that winter in the northeast Atlantic. We use CMR data to implement Cormack‐Jolly‐Seber (CJS) models and estimate adult overwinter survival, while controlling for variation in resighting effort. We relate these estimates to remotely sensed storm data geographically determined by tracking non‐breeding movements with geolocator‐loggers (for two species). These three Alcids are pursuit divers feeding primarily on shoaling fish (e.g. sandeels *Ammodytes* spp. and sprat *Sprattus sprattus*). They also have high wing loading with consequent high energy flight costs (Elliott et al., 2013) which may explain why they are often found among north Atlantic seabird wrecks (e.g. Morley et al., 2016). Storms are expected to reduce survival in all three species, but species‐specific differences in flight costs and winter ranges may lead to divergent effects (Clairbaux et al., 2021). Conversely, storm impacts may be limited as adult survival is expected to be robust to environmental variability in long‐lived species with slow life histories (Gaillard & Yoccoz, 2003). As well as quantifying the effect of overall winter storms, we explore which storm characteristics (i.e. number, intensity, duration, gap between storms, wave height and wind speed) can signal mortality.

## 2 METHODS

### 2.1 Study species and field methods

Fieldwork was conducted on Skomer Island, Wales, UK (51.74°N, 5.30°E), the breeding site for approximately 35,000 Atlantic puffins (hereafter ‘puffins’), 27,000 common guillemots (‘guillemots’) and 8000 razorbills (Newman et al., 2021). Guillemots and razorbills are primarily cliff‐nesters while puffins nest in burrows on grassy slopes or rocky crevices. Adults of all three species were marked by trained fieldworkers, under licence from the British Trust for Ornithology (BTO) on behalf of the UK Home Office, with individually identifiable metal and plastic leg‐rings, and up to daily searches for ringed birds were made during the breeding season to generate long‐term longitudinal encounter histories at focal sub‐colonies: 1985–2021 for guillemots (Amos), 1970–2021 for razorbills (Tom's House) and 1972–2021 for puffins (Isthmus). A total of 798 guillemots, 1282 puffins and 689 razorbills were caught and marked as adults, with 709, 1151 and 597 individuals resighted at least once, resulting in a total of 5877, 3975 and 7729 encounters, respectively (Data S1). Fieldwork was conducted with approval from the Wildlife Trust of South and West Wales.

### 2.2 Winter distributions

We identified puffin and guillemot wintering areas using geolocators during 2007–2014 and 2009–2013, respectively (Supplement S1; Fayet et al., 2016). In total, 31 migratory tracks were obtained from 22 individual guillemots and 109 tracks from 54 puffins. We do not have tracking data for razorbills, but we assume their wintering range is similar to guillemots since ring recoveries and tracking studies from elsewhere suggest their wintering ranges are comparable (Buckingham et al., 2022; Harris & Swann, 2002; Merne, 2002). We focussed on their core wintering period during December–February (meteorological winter) to select environmental covariates, as this is when the strongest storms occur on average and are expected to have the greatest impacts on adult survival. Full processing details of tracking data are available in Supplement S1.

**FIGURE 1:**
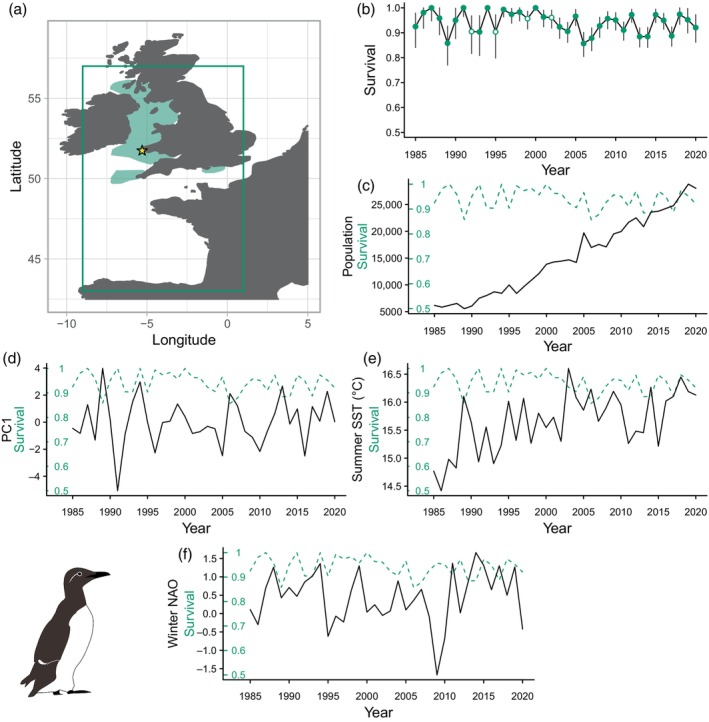
(a) Winter 90% utilisation distribution (green polygon) of common guillemots breeding on Skomer Island (yellow star)—green box shows extent covariates were extracted from. (b) Time‐dependent adult survival probabilities and 95% confidence intervals, oil spill years are highlighted by points with white centres. (c) Temporal variation in population size, (d) storminess, (e) summer SST and (f) winter NAOI (black solid lines), compared to adult survival (green dashed lines).

**FIGURE 2:**
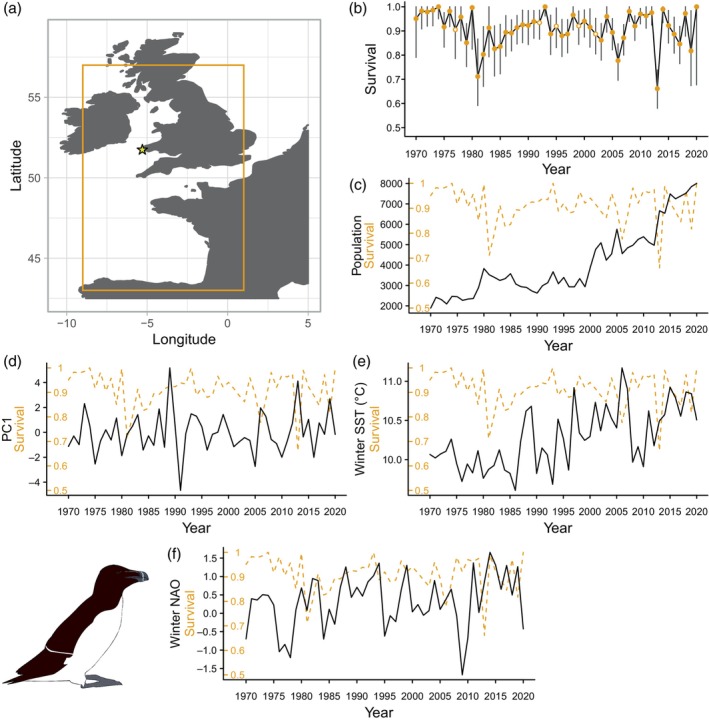
(a) Orange box represents estimated razorbill wintering area for Skomer Island (yellow star). (b) Time‐dependent adult survival probabilities and 95% confidence intervals, oil spill years are highlighted by points with white centres. (c) Temporal variation in population size, (d) storminess, (e) winter SST and (f) winter NAOI (black solid lines), compared to adult survival (orange dashed lines).

**FIGURE 3:**
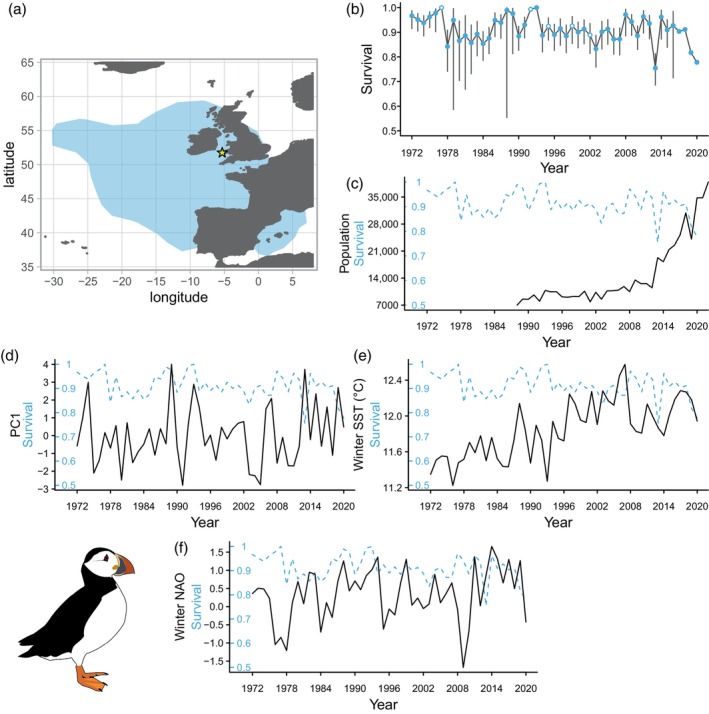
(a) Winter 90% utilisation distribution (blue polygon) of Atlantic puffins breeding on Skomer Island (yellow star). (b) Time‐dependent adult puffin survival probabilities and 95% confidence intervals, oil spill years are highlighted by points with white centres. Confidence intervals for the years 1977, 1992, 1993, 2017, 2019 and 2020 were inestimable and removed for clarity. The plot with full confidence intervals is available as Figure S7. (c) Temporal variation in population size, (d) storminess, (e) winter SST and (f) winter NAOI (black solid lines), compared to adult survival (blue dashed lines).

As tracking data did not span the full range of years of CMR data, we analysed annual consistency in puffin and guillemot winter distributions to test for any changes. Using the R package *adehabitatHR* (Calenge, 2006; R Core Team, 2022), we created annual population‐level 90% utilisation distributions (UD) for both species and calculated the proportion of among‐year overlap. As the 90% UDs showed a high degree of overlap among years in both species (Tables S1 and S2; Figures S3 and S4), all years were pooled (panel a in Figures 1 and 3). The Europe Albers Equal Area Conic projection was used to create UDs and assess similarity, then UDs were reprojected to WGS1984 for mapping. The puffin wintering area fell within −30.25–7.08°E, 36.55–59.4°N and the wintering area of guillemots and razorbills was expanded to the area −9–1°E, 43–57°N (panel a in Figures 1 and 2), as birds ringed in Pembrokeshire are frequently recovered in the Bay of Biscay (Harris & Swann, 2002; Merne, 2002; Votier et al., 2008).

### 2.3 Winter storms

Storm data were obtained from the ERA5 reanalysis from the European Centre for Medium‐Range Weather Forecasting (Hersbach et al., 2020) following the methods outlined in Priestley et al. (2020). Briefly, storms were identified and tracked using 850 hPa relative vorticity (Hoskins & Hodges, 2002), then filtered to retain tracks that lasted over 48 h and travelled over 1000 km to remove short‐lived, stationary and weak storms. The number of days exceeding wind speed and wave height thresholds were also extracted from the ERA5 reanalysis. Wind gusts were defined as the maximum 3 s gust in the last hour at a height of 10 m; and wave height was calculated as the average height between a wave peak and trough. Storm, wind and wave data were extracted for December–February 1985–2021 for guillemots; 1972–2021 for puffins; and 1970–2021 for razorbills. A description of the storm variables, wind speed and wave height thresholds extracted is presented in Table 1.

**TABLE 1:** Description of storm and extreme wind and wave variables extracted and used as covariates in CMR analysis.

Variable	Definition	Justification
Number of storms	Total number passing through the wintering area	Previously found to reduce guillemot and puffin survival (Louzao et al., 2019; Reiertsen et al., 2021)
Mean storm intensity	Mean of maximum storm intensity (minimum sea level pressure) when in the wintering area	Seabirds reduce activity during higher intensity cyclones (Clairbaux et al., 2021)
Mean storm duration	Mean number of hours storms are present in wintering area	Seabirds expected to starve during longer storms (Clairbaux et al., 2021)
Mean gap	Mean time between storms in hours	Many storms in a short period found to increase guillemot mortality (Louzao et al., 2019)
Wave height and wind speed	Number of days exceeding 5/7/10 m wave height & 30/35 m/s wind speed	A representation of sea and therefore foraging conditions

Storm variables covaried strongly and thus could not be combined as individual covariates. Therefore, we transformed all variables into a single component that contained most of the information using a Principal Component Analysis (PCA) with the R package *FactoMineR* (Le et al., 2008). ‘Storminess’ was represented by the first principal component (PC1) extracted from the PCA (Gimenez & Barbraud, 2017), as it was strongly correlated with all storm variables and explained the greatest proportion of variance (Tables S3 and S4). High values of PC1 represented stormy winters, associated with more tightly packed, intense storms frequently exceeding wind speed and wave height thresholds; and low values of PC1 represented calmer winters with fewer, less frequent and weaker storms, exceeding wind speed and wave height thresholds less often (Table S4).

### 2.4 Additional covariates

The NAOI is the pressure difference between the Azores and Iceland with positive indices linked to higher storm activity and reduced seabird survival (e.g. Sandvik et al., 2005; Votier et al., 2005). However, the NAOI may also have indirect impacts, by influencing sea temperature and prey availability (Arnott & Ruxton, 2002). We included mean December to February NAOI values (National Oceanic and Atmospheric Administration (NOAA) Climate Prediction Centre: https://www.cpc.ncep.noaa.gov/products/precip/CWlink/pna/nao.shtml), to determine its efficacy at characterising winter storms.

Warming seas have changed the abundance, distribution and lipid content of some forage fish, reducing seabird breeding success and survival (Sydeman et al., 2021; Wanless et al., 2005). We extracted average summer (June–August; sSST) and winter (December–February; wSST) sea surface temperature from NOAA's extended reconstructed SST V5 (ERSSTv5), a global monthly SST analysis on a 2° × 2° grid (Huang et al., 2017; available from: https://coastwatch.pfeg.noaa.gov/erddap/griddap/nceiErsstv5.html), to account for any impacts on survival arising from variability in food supply. We included both sSST and wSST as reduced food quality or quantity during breeding may have carry‐over impacts on winter condition and the ability to buffer storms, and SST during non‐breeding may impact prey availability and quality. We also included sSST and wSST lagged by 1 year as seabird demographic rates have previously been linked to SST impacts on forage fish life cycles (Frederiksen et al., 2004).

Between 1970 and 2021, five major oil spills (>10,000 t) occurred within the three species' wintering areas: *Amoco Cadiz* (March 1978, Brittany), *Aegean Sea* (December 1992, Galicia), *Sea Empress* (February 1996, Pembrokeshire), *Erika* (December 1999, Brittany) and *Prestige* (November 2002, Galicia); each of which killed or fouled large numbers of seabirds leading to reduced adult guillemot survival, but increased immature recruitment on Skomer (Votier et al., 2005, 2008). We included oil spills as a two‐level factor (oil spill year or not).

As populations of all three species are growing on Skomer, we included annual whole island counts of guillemots and razorbills (Newman et al., 2021), taking the moving average in years with no counts (guillemots: 2016, 2018, 2020; razorbills: 2017, 2019, 2020). Counts represent the total number of breeding and non‐breeding individuals at the colony. Puffin population counts were omitted from CMR models as counts began only in 1988.

### 2.5 Mark‐recapture analysis

#### 2.5.1 Goodness‐of‐fit

Apparent survival (ϕ) and resighting probabilities (p) for each species were estimated from encounter histories using Cormack‐Jolly‐Seber (CJS) models. The goodness‐of‐fit (GOF) of a fully time‐dependent CJS model to the datasets was tested using U‐CARE (Choquet et al., 2020; Table S5); however, all species failed Test 2.CT, indicating trap‐dependence (guillemot: *χ* ^2^_34_ = 1431.80, *p* < 0.001; razorbill: *χ* ^2^_49_ = 1081.21, *p* < 0.001; puffin: *χ* ^2^_47_ = 2350.99, *p* < 0.001). Encounter histories were therefore split at each resighting in U‐CARE following Pradel (1993). Resighting probability was modelled incorporating a trap effect (m), where resighting was modelled separately for individuals sighted at the previous occasion, and those that were not, using a two‐group model structure (Table 2). Accounting for trap effects improved the model fit for all three species, and remaining lack of fit was accounted for by including a variance inflation factor (*ĉ*) of 1.475 for guillemots, 2.307 for puffins, and 1.493 for razorbills (White & Burnham, 1999). Although both components of Test 3 were also statistically significant for puffins (Test 3.SR: *χ* ^2^_48_ = 80.366, *p* = 0.002; Test 3.SM: *χ* ^2^_53_ = 89.021, *p* < 0.001), the *ĉ* was <3 (White & Burnham, 1999; Table S5), so lack of fit was accounted for through the variance inflation factor.

**TABLE 2:** Candidate resighting (p) model structures tested for (a) guillemots, (b) razorbills and (c) puffins.

Model	QAICc	ΔQAICc	QAICc weights	Model likelihood	Num. par	QDeviance
(a) Guillemot						
**ϕ**_**t**_ **p**_.**/t**_	**5717.600**	**0**	**0.715**	**1.000**	**72**	**2656.833**
ϕ_**t**_ p_t/._	5720.182	2.582	0.197	0.275	73	2657.369
ϕ_**t**_ p_t + m_	5721.772	4.171	0.089	0.124	74	2656.911
ϕ_**t**_ p_./._	5814.542	96.942	0	0.000	38	2822.970
(b) Razorbill						
ϕ_**t**_ **p**_**t/**._	**5115.930**	**0**	**1**	**1**	**103**	**2601.345**
ϕ_**t**_ p_t + m_	5163.051	47.121	0	0	104	2646.372
ϕ_**t**_ p_./t_	5205.476	89.546	0	0	102	2692.983
ϕ_**t**_ p_./._	5226.406	110.476	0	0	53	2815.320
(c) Puffin						
ϕ_**t**_ **p**_**t + m**_	**7394.549**	**0**	**1**	**1**	**100**	**3234.068**
ϕ_**t**_ p_t/._	7424.594	30.045	0	0	99	3266.159
ϕ_**t**_ p_./t_	7500.792	106.244	0	0	98	3344.403
ϕ_**t**_ p_./._	7577.681	183.133	0	0	51	3516.906

*Note*: Subscript definitions are as follows: t = time‐dependent; . = constant over time; t + m = additive time and trap effects (same slope, different intercept); forward slash (/) separates resighting model structure for birds sighted at the previous occasion, and those not sighted at the previous occasion. The best‐fitting models are highlighted in bold.

#### 2.5.2 Modelling process

For each species, we created a candidate set of resighting models and selected the model with the lowest quasi‐likelihood adjusted Akaike's information criterion corrected for small sample sizes (QAICc) as the best‐fitting resighting model (Table 2). We did not include models with an interaction between time and trap‐dependence to avoid overparameterisation (Pradel, 1993).

CJS models including covariates were run for each species in MARK (White & Burnham, 1999) using the *RMark* interface (Laake, 2013). Winter NAOI, sSST, wSST, lagged sSST and wSST, and population size were standardised (mean = 0, SD = 1) and included in models alongside PC1 and oil spills. Population size and all SST variables were strongly positively correlated as all increased over time; therefore, population and SST were not combined in models. We first modelled covariate impacts on survival individually, then combined PC1 with SST covariates, population size, and oil spills in additive and interactive models to create candidate model sets for each species, including models with constant (denoted ‘.’) and time‐dependent (t) survival. We selected the model with the lowest QAICc value as the best‐fitting model and performed analysis of deviance (ANODEV) to determine whether covariates explained a significant amount of variation in survival (Skalski et al., 1993).

We also constrained survival by each storm variable for each species. We also modelled additive and interactive effects of the best‐performing environmental covariate from the overall storminess (PC1) models with the best‐performing storm covariate, selected by lowest QAICc. Finally, we used ANODEV to assess whether storm variables could explain significant proportions of annual variation in survival.

## 3 RESULTS

### 3.1 Description of demographic rates

Populations of all three species increased over the study: guillemots from 6181 individuals in 1985 to 28,033 in 2020 (Figure 1c); razorbills from 1893 individuals in 1970 to 8008 in 2020 (Figure 2c) and puffins from 8537 individuals in 1988 to 34,813 in 2020 (Figure 3c). The range of adult overwinter survival rates were as follows: guillemot: 0.857–1 (Figure 1b; mean and 95% confidence intervals (CI) 0.938, 0.930–0.944), razorbill: 0.661–1 (Figure 2b; mean and 95% CI 0.908, 0.898–0.917) and puffin: 0.761–1 (Figure 3b; mean and 95% CI 0.918, 0.913–0.923), with no clear temporal trends.

### 3.2 Seabird survival, storms and other covariates

**FIGURE 4:**
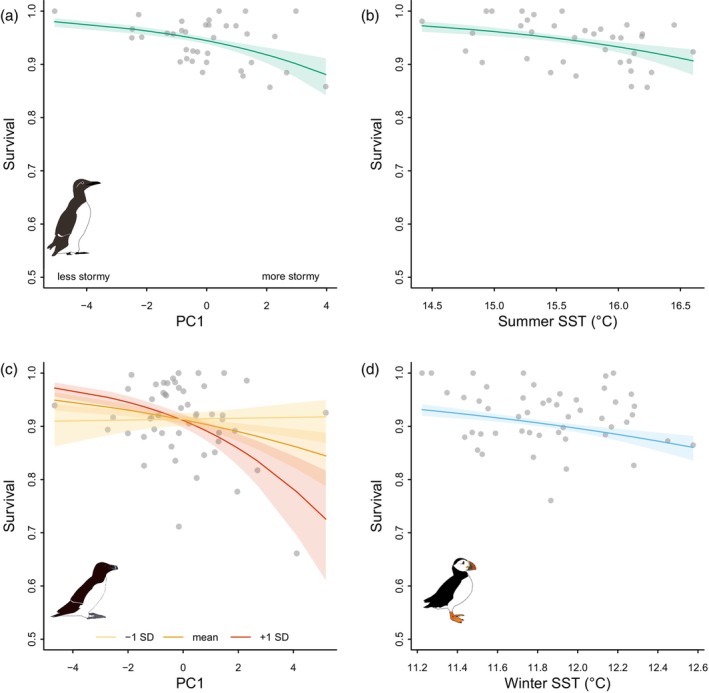
Model predictions (lines) and 95% confidence intervals (shaded areas) of the effect of storminess and other covariates on adult survival of common guillemots (a, b), razorbills (c) and Atlantic puffins (d), from the best covariate model for each species (see Table 3a–c). Grey points show annual survival estimates derived from time‐dependent models, plotted against corresponding covariate values for each year. Plot (c) shows the interaction between storminess and razorbill population (population mean in orange), with the predicted impact of storms for population size one standard deviation smaller than the mean in yellow, and one standard deviation larger than the mean in red.

Guillemot survival was best explained by the time‐dependent model (Table 3a). However, the best covariate model, with additive effects of storminess and sSST, explained a significant proportion of annual variation in survival (ANODEV: 31.5%, *F*_2,33_ = 7.588, *p* = 0.002; Table 3a). Storms and sSST both lowered survival (*β*_PC1_ = −0.210 ± 0.039 standard error, CI = −0.287, −0.133; *β*_sSST_ = −0.313 ± 0.071, −0.453, −0.174; Figure 4a,b). The model including an interaction between storms and sSST performed less well than the additive model (Table 3a).

**TABLE 3:** Ten best‐fitting models and the global model used to estimate annual adult survival for (a) common guillemot, (b) razorbill and (c) Atlantic puffin.

Model	QAICc	ΔQAICc	QAICc weights	Model likelihood	Num. par	QDeviance
(a) Guillemot						
ϕ_t_ p_./t_	5698.712	0	0.868	1	72	2647.827
ϕ_PC1 + sSST_ p_./t_	5703.355	4.643	0.085	0.098	39	2719.640
ϕ_PC1 × sSST_ p_./t_	5705.287	6.575	0.032	0.037	40	2719.547
ϕ_PC1 × population_ p_./t_	5708.755	10.043	0.006	0.007	40	2723.015
ϕ_PC1 + population_ p_./t_	5709.833	11.121	0.003	0.004	39	2726.118
ϕ_PC1 + lag wSST_ p_./t_	5710.800	12.088	0.002	0.002	39	2727.085
ϕ_PC1 + wSST_ p_./t_	5710.878	12.165	0.002	0.002	39	2727.163
ϕ_PC1 × wSST_ p_./t_	5712.276	13.564	0.001	0.001	40	2726.536
ϕ_PC1 × lag wSST_ p_./t_	5712.824	14.112	0.001	0.001	40	2727.084
ϕ_._ p _./t_	5732.333	33.621	0	0	37	2752.667
(b) Razorbill						
ϕ_t_ p_t/._	5115.930	0	0.596	1	103	2601.345
ϕ_PC1 × population_ p_t/._	5117.184	1.254	0.318	0.534	56	2699.952
ϕ_PC1 × lag sSST_ p_t/._	5120.140	4.210	0.073	0.122	56	2702.908
ϕ_PC1 × wSST_ p_t/._	5125.327	9.397	0.005	0.009	56	2708.095
ϕ_PC1 + population_ p_t/._	5126.084	10.154	0.004	0.006	55	2710.902
ϕ_PC1 + wSST_ p_t/._	5127.111	11.182	0.002	0.004	55	2711.930
ϕ_PC1_ p_t/._	5128.945	13.015	0.001	0.002	54	2715.812
ϕ_PC1 + lag wSST_ p_t/._	5129.649	13.720	0.001	0.001	55	2714.468
ϕ_PC1 × lag wSST_ p_t/._	5129.728	13.798	0.001	0.001	56	2712.496
ϕ_._ p_t/._	5137.440	21.511	0	0	53	2726.355
(c) Puffin						
ϕ_wSST_ p_t + m_	7380.130	0	0.364	1	53	3315.306
ϕ_PC1 × lag sSST_ p_t + m_	7381.119	0.989	0.222	0.610	55	3312.245
ϕ_PC1 + wSST_ p_t + m_	7382.154	2.023	0.132	0.364	54	3315.305
ϕ_PC1 × wSST_ p_t + m_	7383.293	3.163	0.075	0.206	55	3314.420
ϕ_lag sSST_ p_t + m_	7383.322	3.192	0.074	0.203	53	3318.499
ϕ_lag wSST_ p_t + m_	7383.468	3.337	0.069	0.189	53	3318.644
ϕ_sSST_ p_t + m_	7385.131	5.001	0.030	0.082	53	3320.308
ϕ_PC1 + lag wSST_ p_t + m_	7385.477	5.347	0.025	0.069	54	3318.629
ϕ_._ p_t + m_	7387.355	7.225	0.010	0.027	52	3324.556
ϕ_t_ p_t + m_	7394.549	14.418	0	0.001	100	3234.068

*Note*: Subscripts represent model structure fitted to survival (ϕ) and resighting (p) probabilities: lag sSST = summer sea surface temperature with 1 year lag; lag wSST = winter sea surface temperature with 1 year lag; PC1 = storminess; population = population size; sSST = summer sea surface temperature; wSST = winter sea surface temperature.

Although the time‐dependent model was the best supported model of razorbill survival, the ΔQAICc of the best covariate model was <2 (Table 3b). This model included interactive effects of storms and population size and explained a significant proportion of variation in annual survival (21.1%, *F*_3,47_ = 4.195, *p* = 0.010). Survival was reduced in stormier years, and this effect was greater at large population sizes (*β*_PC1_ = −0.126 ± 0.036, −0.197, −0.054; *β*_population_ = −0.016 ± 0.061, −0.135, 0.103, *β*_PC1 × population_ = −0.136 ± 0.033, −0.201, −0.071; Figure 4c).

Puffin survival was best explained by wSST (Table 3c), which reduced survival (*β*_wSST_ = −0.187 ± 0.034, −0.266, −0.108) and explained a significant proportion of annual variation (10.2%, *F*_1,47_ = 5.351, *p* = 0.025; Figure 4d). Despite this, the lowest annual value of puffin survival coincided with the highest value of storm intensity (2013/14, Figure 3d).

### 3.3 Impact of different storm characteristics

**FIGURE 5:**
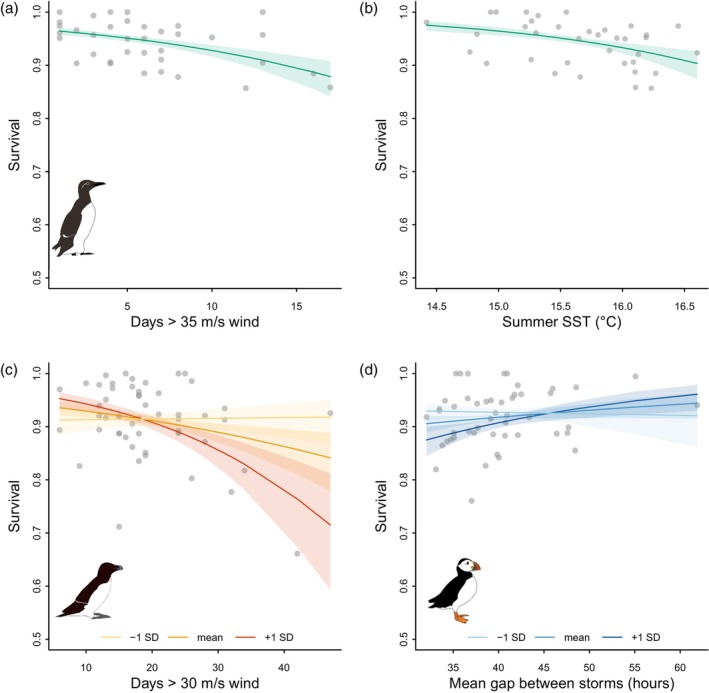
Impact of storm characteristics on auk survival. Model predictions (lines) and 95% confidence intervals (shaded areas) of storm covariate effects on adult survival of common guillemots (a, b), razorbills (c) and Atlantic puffins (d), from the best‐performing model for each species (see Table 4a–c). Grey points show annual survival estimates. Plot (c) shows the interaction between the number of days with wind speeds >30 m/s and population size (population mean in orange). Plot (d) shows the interaction between the mean gap between storms and winter SST (wSST mean in mid‐blue).

In guillemots, the time‐dependent model had the lowest QAICc; however, the ΔQAICc of the model with additive impacts of the number of days per winter with wind speeds exceeding 35 m/s and sSST was 0.15 (Table 4a). This model explained a significant amount of variation in annual survival (35.8%, *F*_2,33_ = 9.196, *p* < 0.001), and wind and sSST both reduced survival (*β*_days>35m/s wind_ = −0.371 ± 0.057, −0.463, −0.239, *β*_sSST_ = −0.352 ± 0.077, −0.503, −0.201; Figure 5a,b).

**TABLE 4:** Impact of storm variables on adult survival for (a) common guillemot, (b) razorbill and (c) Atlantic puffin.

Model	QAICc	ΔQAICc	QAICc weights	Model likelihood	Num. par	QDeviance
(a) Guillemot						
ϕ_t_ p_./t_	5698.712	0	0.426	1	72	2647.827
ϕ_days>35 m/s wind + sSST_ p_./t_	5698.862	0.150	0.395	0.928	39	2715.147
ϕ_days>35 m/s wind × sSST_ p_./t_	5700.456	1.744	0.178	0.418	40	2714.716
ϕ_days>35 m/s wind_ p_./t_	5711.982	13.269	0.001	0.001	38	2730.291
ϕ_days>5m wave_ p _/t_	5712.380	13.668	0	0.001	38	2730.690
ϕ_mean gap between storms_ p_./t_	5722.594	23.882	0	0	38	2740.904
ϕ_mean storm duration_ p_./t_	5730.139	31.427	0	0	38	2748.449
ϕ_total number storms_ p_./t_	5731.517	32.805	0	0	38	2749.826
ϕ_mean storm intensity_ p_./t_	5731.929	33.217	0	0	38	2750.239
ϕ_._ p_./t_	5732.333	33.621	0	0	37	2752.667
(b) Razorbill						
ϕ_days>30 m/s wind × population_ p_t/._	5111.843	0	0.878	1	56	2694.612
ϕ_t_ p_t/._	5115.930	4.086	0.114	0.130	103	2601.345
ϕ_days>30 m/s wind + population_ p_t/._	5121.761	9.918	0.006	0.007	55	2706.579
ϕ_days>30 m/s wind_ p_t/._	5125.010	13.167	0.001	0.001	54	2711.877
ϕ_days>10m wave_ p_t/._	5128.083	16.239	0	0	54	2714.949
ϕ_mean gap between storms_ p_t/._	5133.796	21.953	0	0	54	2720.663
ϕ_total number storms_ p_t/._	5136.065	24.221	0	0	54	2722.931
ϕ_mean storm intensity_ p_t/._	5137.065	25.222	0	0	54	2723.932
ϕ_._ p_t/._	5137.440	25.597	0	0	53	2726.355
ϕ_mean storm duration_ p_t/._	5138.857	27.013	0	0	54	2725.723
(c) Puffin						
ϕ_mean gap between storms × wSST_ p_t + m_	7378.656	0	0.510	1	55	3309.783
ϕ_mean gap between storms + wSST_ p_t + m_	7378.915	0.259	0.448	0.879	54	3312.067
ϕ_mean gap between storms_ p_t + m_	7385.611	6.955	0.016	0.031	53	3320.787
ϕ_._ p_t + m_	7387.355	8.699	0.007	0.013	52	3324.556
ϕ_total number storms_ p_t + m_	7387.927	9.270	0.005	0.010	53	3323.103
ϕ_days>7m wave_ p_t + m_	7387.967	9.311	0.005	0.010	53	3323.144
ϕ_days>30 m/s wind_ p_t + m_	7388.269	9.613	0.004	0.008	53	3323.446
ϕ_mean storm intensity_ p_t + m_	7388.790	10.134	0.003	0.006	53	3323.966
ϕ_mean storm duration_ p_t + m_	7389.142	10.486	0.003	0.005	53	3324.319
ϕ_t_ p_t + m_	7394.549	15.892	0	0	100	3234.068

*Note*: Subscripts represent model structure fitted to survival (ϕ) and resighting (p) probabilities. Variable definitions can be found in Table 1.

Razorbill survival was best explained by the interactive effects of the number of days per winter exceeding 30 m/s wind speed and population size, which accounted for 25.4% of annual variation in survival and was significant (*F*_3,47_ = 5.332, *p* = 0.003; Table 4b). Wind had a negative impact on survival, which was greater at larger population sizes (*β*_days>30m/s wind_ = −0.202 ± 0.061, −0.321, −0.083, *β*_population_ = −0.029 ± 0.058, −0.144, 0.085, *β*_days>30m/s wind × population_ = −0.216 ± 0.052, −0.317, −0.115; Figure 5c). This model had the lowest QAICc of all models of razorbill survival (Tables 3b and 4b).

The best model of puffin survival included interactive effects of the mean gap between storms and wSST, which explained a significant amount of annual variation in survival (16.3%, *F*_3,45_ = 2.927, *p* = 0.044; Table 4c). Puffin survival was higher when the mean gap between storms was longer, while wSST had a negative impact on survival, which was stronger when the mean gap between storms was short (*β*_mean gap_ = 0.110 ± 0.061, −0.009, 0.229, *β*_wSST_ = −0.129 ± 0.044, −0.214, −0.044, *β*_mean gap × wSST_ = 0.135 ± 0.057, 0.022, 0.247; Figure 5d). This model also had the lowest QAICc of all models of puffin survival (Tables 3c and 4c).

## 4 DISCUSSION

We found that winter storms had divergent negative effects on overwinter survival of three seabirds breeding at Skomer, but there was no relationship with NAOI. Adult guillemot and razorbill survival was reduced under stormy conditions, with additive negative effects of sSST for guillemots and stronger storm effects at large population sizes for razorbills. Puffins were negatively impacted by higher wSST, and while there was no relationship with overall storminess, the winter with highest mortality (0.755 during 2013/14) coincided with high storm intensity and a major seabird wreck (Morley et al., 2016). When considering the impact of storm features separately, razorbill and guillemot survival was negatively correlated with the number of days per winter with wind speeds exceeding 30 and 35 m/s, respectively, while puffin survival was positively correlated with the mean gap duration between storms. These effects came against a backdrop of rapid population growth in these three species.

### 4.1 General patterns of auk demography

As expected for adult seabirds, survival rates were generally high. Guillemot survival (0.938) averaged highest of the three species, followed by puffins (0.918) and razorbills (0.908), which is comparable with estimates from these species at other North Atlantic colonies (Grosbois et al., 2009; Lavers et al., 2008; Sandvik et al., 2005).

Populations of all three species grew strongly on Skomer, in marked contrast with declines at other North Atlantic colonies (Bennett, 2023; Buckingham, 2023; Owen et al., 2023)—indeed, puffin population declines mean they are currently classified as Endangered in Europe (BirdLife International, 2021). Auk populations on Skomer are in a period of recovery post‐WW2 following persecution and chronic oiling, with an estimated ~100,000 individual guillemots on Skomer in the 1930s and reports of 40,000 puffins during the 1940s (Birkhead, 2016; Pritchard et al., 2021).

### 4.2 Impact of winter storms on adult survival

Using our overwinter composite storm covariate, we found a negative effect of storms on adult survival for guillemots and razorbills, but not puffins. Storms most likely lowered guillemot and razorbill survival by impeding winter foraging (Birkhead, 1976; Clairbaux et al., 2021) and reducing prey availability (Bacheler et al., 2019), resulting in starvation. We also found an additive, negative effect of sSST on guillemots, possibly caused by reduced breeding season food quality or quantity (Wanless et al., 2005), with downstream consequences for winter condition (Bogdanova et al., 2011). As sea temperatures and storm frequency and intensity are predicted to increase, this may well represent worsening conditions for guillemots.

For razorbills, survival was negatively impacted by storms and this effect was greater as the population grew. The density‐dependent impact on survival is likely a carry‐over effect from the breeding season when intraspecific competition for resources (i.e., food and breeding sites) may reduce body condition and increase susceptibility to winter storms (Wanless et al., 2023).

Despite puffins comprising large proportions of winter seabird wrecks in the north Atlantic (Morley et al., 2016) we found no links between adult survival and storms. It is unclear whether this is due to our inability to detect an effect on survival, or because puffins are not impacted by storms as their very large non‐breeding distribution (Figure 3a; Fayet et al., 2016) means they can move away from storms, or some combination of these factors. Nevertheless, the lowest survival estimate (0.755) in winter 2013/14 coincided with the second highest winter storm index, and when 50,000 auks were found dead along the Atlantic coastlines of Spain, France and the UK (Morley et al., 2016). Reiertsen et al. (2021) also found no influence of extreme extratropical cyclones on puffin survival across four northeast Atlantic colonies, except for one Norwegian population exposed to the most extreme weather. Therefore, perhaps only the most extreme storms negatively impact adult puffin survival, suggesting that non‐linear impacts or tipping points associated with climate extremes require more investigation (Oro, 2014). There is evidence for non‐linear effects of extremes in sea ice extent on polar seabird demographics (e.g. Christie et al., 2018), and guillemot mortality is related to cumulative frequency of extreme wind events in the Bay of Biscay (Louzao et al., 2019). Although some of the lowest survival estimates coincided with high values of PC1, our models were constrained by environmental covariates in a linear framework, which likely inhibited detection of non‐linear storm impacts in this study.

Despite years with low survival rates (the 2013/14 estimates for razorbills [0.661] and puffins [0.755] are among the lowest values recorded for seabirds; Sydeman et al., 2022), their breeding populations continued to increase, possibly due to increased recruitment of immigrant or natal immatures in response to newly vacant breeding sites (Votier et al., 2008). More research is needed to understand climatic drivers of immature seabird demography and their long‐term ability to buffer impacts on the breeding population.

Other multi‐species studies of climate change impacts on seabirds found a lack of generality (e.g. Clucas et al., 2014), consistent with our results. Nevertheless, it was the strength of the storm effect as well as other climate indices which appeared to drive this variation in our study. For instance, wSST had the greatest impact on puffin survival, which is presumably linked with overwinter food availability; however, guillemots and razorbills were instead more impacted by winter storms. The reasons for such differences are unclear but may relate to physiological differences related to body size and wing‐loading variation (Elliott et al., 2013); or extended parental care beyond colony departure in guillemots and razorbills, which also coincides with adults' moulting period (St John Glew et al., 2018).

When winter storms were broken down into individual components, the number of days where wind speed exceeded 30 and 35 m/s had negative impacts on razorbill and puffin survival, respectively; while puffin survival was positively correlated with the mean gap between storms. These effects most likely arise because of impacts on foraging conditions (Clairbaux et al., 2021), but such storm characteristics might form a more effective predictive tool than storms alone and provide clearer mechanisms than wide‐scale climate indices such as the NAOI. These results also highlight interspecific differences in storm vulnerability—guillemots and razorbills may be particularly at risk from projected increases in frequency of the most intense storms for the coastal regions surrounding northwest Europe (Priestley & Catto, 2022).

### 4.3 Methodological remarks

We could perform only a partial test of the assumption that puffin and guillemot winter distribution had not changed over time because tracking data covered only a short span of the CMR data. However, the storm and environmental covariates we chose to extract from the wintering range selected appear appropriate as the best models include these covariates.

We are confident our results are robust as our data meet the underlying assumptions of CMR modelling, and we accounted for the lack of fit arising from trap‐dependence. Birds were ringed as breeding adults, which display high breeding site philopatry (88%–97%; Ashcroft, 1979; Harris et al., 1996; Lavers et al., 2007), so all individuals should have equal survival probability, and dispersal should be minimal. Birds were marked with metal rings combined with plastic colour rings designed to facilitate resighting from distance and withstand abrasion, so it is unlikely any marks were missed or lost. Further, most birds seen or ringed on each occasion were subsequently resighted (Data S1). However, resighting effort varied among species, resulting in lower resighting probabilities in razorbills and puffins than guillemots (Figures S6–S8) which may have hampered our ability to detect storm impacts in puffins. Finally, although senescence and sex differences in survival have previously been reported in seabirds (e.g. Gianuca et al., 2019; Landsem et al., 2023), we could not consider these in our analyses as the age and sex at ringing of most birds was unknown.

## 5 CONCLUSIONS

Winter storms are a common feature of temperate marine ecosystems, and we found differing signs of their deleterious impact, combined with effects of warming seas and density‐dependence, among three diving seabirds in the northeast Atlantic. We identified extended periods with strong winds as a possible storm characteristic predictive of guillemot and razorbill mortality, while puffin mortality may be elevated by storms occurring in rapid succession. It is unclear why these closely related sympatric breeders responded differently to storms, but it may relate to different migratory strategies or energetics. We also found evidence that puffin survival was lowest only under the most intense storms, which also coincided with a major seabird wreck—pointing towards non‐linear effects. Despite this, all three species showed rapid population increases, likely due to recovery from long‐term persecution. Therefore, while the trajectories suggest little population‐level impact, as storm frequency and intensity increase, the seas continue to warm, and density‐dependent effects establish themselves, winter wrecks may have more profoundly negative consequences in future. Detailed longitudinal population monitoring is therefore vitally important in future, alongside quantifying storm impacts on the large number of non‐breeders that comprise a major proportion of seabird populations.

## AUTHOR CONTRIBUTIONS

Kirsty Laurenson, Stephen C. Votier and Richard B. Sherley conceived the ideas and designed methodology; Matt J. Wood, Tim R. Birkhead and Ben J. Hatchwell acquired and provided auk demographic data; Tim R. Birkhead and Ben J. Hatchwell maintain and manage the long‐term study of guillemots on Skomer; Tim R. Birkhead, Annette L. Fayet and Tim Guilford acquired and provided guillemot and puffin tracking data; Matthew D. K. Priestley acquired and provided storm data; Kirsty Laurenson analysed the data; Kirsty Laurenson and Stephen C. Votier led writing of the manuscript. All authors contributed critically to the drafts and gave final approval for publication.

## CONFLICT OF INTEREST STATEMENT

The authors declare no conflicts of interest.

## Supporting information


**Figure S1:** Example immersion data plot used to determine timing of calibration periods.
**Figure S2:** Example plot of raw light data from geolocators, which visualises timing of twilights recorded by GLS devices over time.
**Figure S3:** Annual winter 90% UDs of all adult guillemots tracked.
**Figure S4:** Annual winter 90% UD of all adult puffins tracked.
**Figure S5:** Annual survival estimates and 95% profile likelihood confidence intervals for Atlantic puffins from the model ϕ_t_ p_t/t_.
**Figure S6:** Annual resighting estimates of common guillemots, from the model ϕ_t_ *p*_./t_.
**Figure S7:** Annual resighting rate of razorbills, from the model ϕ_t_ *p*_t/._.
**Figure S8:** Annual resighting rate of Atlantic puffins, from the model ϕ_t_ *p*_t + m_.
**Table S1:** Proportion of each annual 90% utilisation distribution (UD) that overlapped with the UD of another year.
**Table S2:** Proportion of each annual 90% UD that overlapped with the UD of another year.
**Table S3:** Results of PCA performed on storm variables extracted from each species' wintering area.
**Table S4:** Storm variables significantly correlated with PC1 and the direction of the relationship.
**Table S5:** Goodness of fit test results for guillemots, razorbills, and puffins.

**Data S1:** Capture histories for common guillemots, razorbills, and Atlantic puffins summarised in the m‐array format.

## Data Availability

Guillemot demographic data and environmental covariates are freely available from Heriot Watt research portal https://doi.org/10.17861/8fd6a194‐d1a7‐469c‐8604‐e45b0414f8f0 (Laurenson et al., 2024a). Puffin and razorbill demographic data are available upon request for non‐commercial use from the University of Gloucester online repository https://doi.org/10.46289/8FP68KK9 (Laurenson et al., 2024b).

## References

[jane14227-bib-0001] Arnott, S. A. , & Ruxton, G. D. (2002). Sandeel recruitment in the North Sea: Demographic, climatic and trophic effects. Marine Ecology Progress Series, 238, 199–210. 10.3354/meps238199

[jane14227-bib-0002] Ashcroft, R. E. (1979). Survival rates and breeding biology of puffins on Skomer Island, Wales. Ornis Scandinavica, 10, 100–110. 10.2307/3676349

[jane14227-bib-0003] Bacheler, N. M. , Shertzer, K. W. , Cheshire, R. T. , & MacMahan, J. H. (2019). Tropical storms influence the movement bahviour of a demersal oceanic fish species. Scientific Reports, 9, 1481. 10.1038/s41598-018-37527-1 30728378 PMC6365635

[jane14227-bib-0004] Bennett, S. (2023). Common guillemot *Uria aalge* . In D. Burnell , A. J. Perkins , S. E. Newton , M. Bolton , T. D. Tierney , & T. E. Dunn (Eds.), Seabirds count: A census of breeding seabirds in Britain and Ireland (2015–2021) (pp. 367–382). Lynx Nature Books.

[jane14227-bib-0005] BirdLife International . (2021). *Fratercula arctica* (Europe assessment). The IUCN Red List of threatened species 2021. e.T22694927A166290968. 10.2305/IUCN.UK.2021-3.RLTS.T22694927A166290968.en

[jane14227-bib-0006] Birkhead, T. R. (1976). Effects of sea conditions on rates at which guillemots feed chicks. British Birds, 69, 490–492.

[jane14227-bib-0007] Birkhead, T. R. (2016). Changes in the number of common guillemots on Skomer since the 1930s. British Birds, 109, 651–659.

[jane14227-bib-0008] Boano, G. , Brichetti, P. , & Foschi, U. F. (2010). La‐Nina driven Atlantic storms affect winter survival of Mediterranean Cory's shearwaters. Italian Journal of Zoology, 77, 460–468. 10.1080/11250000903469017

[jane14227-bib-0009] Bogdanova, M. , Daunt, F. , Newell, M. , Phillips, R. A. , Harris, M. P. , & Wanless, S. (2011). Seasonal interactions in the black legged kittiwake *Rissa tridactyla*: Links between breeding performance and winter distribution. Proceedings of the Royal Society B, 278, 2412–2418. 10.1098/rspb.2010.2601 21208952 PMC3125632

[jane14227-bib-0010] Buckingham, L. (2023). Razorbill *Alca torda* . In D. Burnell , A. J. Perkins , S. E. Newton , M. Bolton , T. D. Tierney , & T. E. Dunn (Eds.), Seabirds count: A census of breeding seabirds in Britain and Ireland (2015–2021) (pp. 383–396). Lynx Nature Books.

[jane14227-bib-0011] Buckingham, L. , Bogdanova, M. I. , Green, J. A. , Dunn, R. E. , Wanless, S. , Bennett, S. , Bevan, R. M. , Call, A. , Canham, M. , Corse, C. J. , Harris, M. P. , Heward, C. J. , Jardine, D. C. , Lennon, J. , Parnaby, D. , Redfern, C. P. F. , Scott, L. , Swann, R. L. , Ward, R. M. , … Daunt, F. (2022). Interspecific variation in non‐breeding aggregation: A multi‐colony tracking study of two sympatric seabirds. Marine Ecology Progress Series, 684, 181–197. 10.3354/meps13960

[jane14227-bib-0012] Calenge, C. (2006). The package ‘adehabitat’ for the R software: A tool for the analysis of space and habitat use by animals. Ecological Modelling, 197, 516–519. 10.1016/j.ecolmodel.2006.03.017

[jane14227-bib-0013] Choquet, R. , Lebreton, J.‐D. , Giminez, O. , Reboulet, A. M. , & Pradel, R. (2020). U‐CARE 3.3 User's manual. CEFE. https://www.cefe.cnrs.fr/fr/recherche/bc/bbp/264‐logiciels

[jane14227-bib-0014] Christie, K. , Hollmen, T. , Flint, P. , & Douglas, D. (2018). Non‐linear effect of sea ice: Spectacled eider survival declines at both extremes of the ice spectrum. Ecology and Evolution, 8, 11808–11818. 10.1002/ece3.4637 30598778 PMC6303746

[jane14227-bib-0015] Clairbaux, M. , Mathewson, P. , Porter, W. , Fort, J. , Strom, H. , Moe, B. , Fauchald, P. , Descamps, S. , Helgason, H. H. , Brathen, V. S. , Merkel, B. , Anker‐Nilssen, T. , Bringsvor, I. S. , Chastel, O. , Christensen‐Dalsgaard, S. , Danielsen, J. , Daunt, F. , Dehnhard, N. , Erikstad, K. E. , … Gremillet, D. (2021). North Atlantic winter cyclones starve seabirds. Current Biology, 31, 3964–3971. 10.1016/j.cub.2021.06.059 34520704

[jane14227-bib-0016] Clucas, G. V. , Dunn, M. J. , Dyke, G. , Emslie, S. D. , Levy, H. , Naveen, R. , Polito, M. J. , Pybus, O. G. , Rogers, A. D. , & Hart, T. (2014). A reversal of fortunes: Climate change ‘winners’ and ‘losers’ in Antarctic peninsula penguins. Scientific Reports, 4, 5024. 10.1038/srep05024 24865774 PMC4034736

[jane14227-bib-0017] Costa, D. P. , Breed, G. A. , & Robinson, P. W. (2012). New insights into pelagic migrations: Implications for ecology and conservation. Annual Review of Ecology, Evolution, and Systematics, 43, 73–96. 10.1146/annurev-ecolsys-102710-145045

[jane14227-bib-0018] Dias, M. P. , Martin, R. , Pearmain, E. J. , Burfield, I. J. , Small, C. , Phillips, R. A. , Yates, O. , Lascelles, B. , Borboroglu, P. G. , & Croxall, J. P. (2019). Threats to seabirds: A global assessment. Biological Conservation, 237, 525–537. 10.1016/j.biocon.2019.06.033

[jane14227-bib-0019] Diffenbaugh, N. , Singh, D. , Mankin, J. , Horton, D. , Swain, D. , Tourma, D. , Charland, A. , Liu, Y. , Haugen, M. , Tsiang, M. , & Rajaratnam, B. (2017). Quantifying the influence of global warming on unprecedented extreme climate events. Proceedings of the National Academy of Sciences, 114, 4881–4886. 10.1073/pnas.1618082114 PMC5441735 28439005

[jane14227-bib-0020] Dobson, F. S. , & Jouventin, P. (2007). How slow breeding can be selected in seabirds: Testing Lack's hypothesis. Proceedings of the Royal Society B, 274, 275–279. 10.1098/rspb.2006.3724 17148257 PMC1685855

[jane14227-bib-0021] Elliott, K. H. , Ricklefs, R. E. , Gaston, A. J. , Hatch, S. A. , Speakman, J. R. , & Davoren, G. K. (2013). HIgh flight costs, but low dive costs, in auks support the biomechanical hypothesis for flightlessness in pengiuns. Proceedings of the National Academy of Sciences, 110, 9380–9384. 10.1073/pnas.1304838110 PMC3677478 23690614

[jane14227-bib-0022] Fayet, A. L. , Freeman, R. , Shoji, A. , Boyle, D. , Kirk, H. L. , Dean, B. J. , Perrins, C. M. , & Guilford, T. (2016). Drivers and fitness consequences of dispersive migration in a pelagic seabird. Behavioural Ecology, 27, 1061–1072. 10.1093/beheco/arw013 PMC4943109 27418752

[jane14227-bib-0023] Frederiksen, M. , Wanless, S. , Harris, M. P. , Rothery, P. , & Wilson, L. J. (2004). The role of industrial fisheries and oceanographic change in the decline of North Sea black‐legged kittiwakes. Journal of Applied Ecology, 41, 1129–1139. 10.1111/j.0021-8901.2004.00966.x

[jane14227-bib-0024] Gaillard, J. M. , & Yoccoz, N. G. (2003). Temporal variation in survival of mammals: A case of environmental canalization? Ecology, 84, 3294–3306. 10.1890/02-0409

[jane14227-bib-0025] Genovart, M. , Sanz‐Aguilar, A. , Fernandez‐Chacon, A. , Igual, J. M. , Pradel, R. , Forero, M. G. , & Oro, D. (2013). Contrasting effects of climatic variability on the demography of a trans‐equatorial migratory seabird. Journal of Animal Ecology, 82, 121–130. 10.1111/j.1365-2656.2012.02015.x 22823099

[jane14227-bib-0026] Gianuca, D. , Votier, S. C. , Pardo, D. , Wood, A. G. , Sherley, R. B. , Ireland, L. , Choquet, R. , Pradel, R. , Townley, S. , Forcada, J. , Tuck, G. N. , & Phillips, R. A. (2019). Sex‐specific effects of fisheries and climate on the demography of sexually dimorphic seabirds. Journal of Animal Ecology, 88, 1366–1378. 10.1111/1365-2656.13009 31187479

[jane14227-bib-0027] Gimenez, O. , & Barbraud, C. (2017). Dealing with many correlated covariates in capture‐recapture models. Population Ecology, 59, 287–291. 10.1007/s10144-017-0586-1

[jane14227-bib-0028] Grosbois, V. , Harris, M. P. , Anker‐Nilssen, T. , McCleery, R. H. , Shaw, D. N. , Morgan, B. J. T. , & Giminez, O. (2009). Modeling survival at multi‐population scales using mark‐recapture data. Ecology, 90, 2922–2932. 10.1890/08-1657.1 19886500

[jane14227-bib-0029] Guery, L. , Descamps, S. , Hodges, K. I. , Pradel, R. , Moe, B. , Hanssen, S. A. , Erikstad, K. E. , Gabrielsen, G. W. , Gilchrist, H. G. , Jenouvrier, S. , & Bety, J. (2019). Winter extratropical cyclone influence on seabird survival: Variation between and within common eider *Somateria mollissima* populations. Marine Ecology Progress Series, 627, 155–170. 10.3354/meps13066

[jane14227-bib-0030] Harris, M. P. , & Swann, B. (2002). Common guillemot (Guillemot) *Uria aalge* . In C. Wernham , M. Toms , J. H. Marchant , J. Clark , G. Siriwardena , & S. R. Baillie (Eds.), The migration atlas: Movements of the birds of Britain and Ireland (pp. 397–400). T. & A.D. Poyser.

[jane14227-bib-0031] Harris, M. P. , Wanless, S. , & Barton, T. R. (1996). Site use and fidelity in the common guillemot *Uria Aalge* . Ibis, 138, 399–404. 10.1111/j.1474-919X.1996.tb08057.x

[jane14227-bib-0032] Hersbach, H. , Bell, B. , Berrisford, P. , Hirahara, S. , Horányi, A. , Muñoz‐Sabater, J. , Nicolas, J. , Peubey, C. , Radu, R. , Schepers, D. , & Simmons, A. (2020). The ERA5 global reanalysis. Quarterly Journal of the Royal Meteorological Society, 146, 1999–2049. 10.1002/qj.3803

[jane14227-bib-0033] Hoskins, B. J. , & Hodges, K. I. (2002). New perspectives on the northern hemisphere winter storm tracks. Journal of the Atmospheric Sciences, 59, 1041–1061. 10.1175/1520-0469(2002)059<1041:NPOTNH>2.0.CO;2

[jane14227-bib-0034] Huang, B. , Thorne, P. B. , Banzon, V. F. , Boyer, T. , Chepurin, G. , Lawrimore, J. H. , Menne, M. J. , Smith, T. M. , Vose, R. S. , & Zhang, H.‐M. (2017). NOAA extended reconstructed sea surface temperature (ERSST), version 5 . [1984‐01‐01–2021‐01‐31, 49–57°N, −1–8°E].

[jane14227-bib-0035] Hurrell, J. (1995). Decadal trends in the North Atlantic oscillation: Regional temperatures and precipitation. Science, 269, 676–679. 10.1126/science.269.5224.676 17758812

[jane14227-bib-0036] Kossin, J. , Knapp, K. , Olander, T. , & Velden, C. (2020). Global increase in major tropical cyclone exceedance probability over the past four decades. National Academy of Sciences of the United States of America, 117, 1195–11980. 10.1073/pnas.1920849117 PMC7275711 32424081

[jane14227-bib-0037] Laake, J. L. (2013). RMark: An R interface for analysis of capture‐recapture data with MARK . AFSC Processed Rep 2013‐01, Alaska Fish. Sci. Cent., NOAA, Natl. Mar. Fish. Serv., 7600 Sand Point Way NE, Seattle WA 98115.

[jane14227-bib-0038] Landsem, T. L. , Yoccoz, N. G. , Layton‐Matthews, K. , Hilde, C. H. , Harris, M. P. , Wanless, S. , Daunt, F. , Reiertsen, T. K. , Erikstad, K. E. , & Anker‐Nilssen, T. (2023). Raising offspring increases ageing: Differences in senescence among three populations of a long‐lived seabird, the Atlantic puffin. Journal of Animal Ecology, 92, 774–785. 10.1111/1365-2656.13884 36633069

[jane14227-bib-0039] Laurenson, K. , Wood, M. J. , Birkhead, T. R. , Priestley, M. D. K. , Sherley, R. B. , Fayet, A. L. , Guilford, T. , Hatchwell, B. J. , & Votier, S. C. (2024a). Data from: Long‐term multi‐species demographic studies reveal divergent negative impacts of winter storms on seabird survival. *Heriot Watt Repository*, 10.17861/8fd6a194-d1a7-469c-8604-e45b0414f8f0 PMC11729536 39562515

[jane14227-bib-0040] Laurenson, K. , Wood, M. J. , Birkhead, T. R. , Priestley, M. D. K. , Sherley, R. B. , Fayet, A. L. , Guilford, T. , Hatchwell, B. J. , & Votier, S. C. (2024b). Data from: Long‐term multi‐species demographic studies reveal divergent negative impacts of winter storms on seabird survival. *University of Gloucester Repository*, 10.46289/8FP68KK9 PMC11729536 39562515

[jane14227-bib-0041] Lavers, J. L. , Jones, I. L. , & Diamond, A. W. (2007). Natal and breeding dispersal of razorbills (*Alca torda*) in eastern North America. Waterbirds, 30, 588–594.

[jane14227-bib-0042] Lavers, J. L. , Jones, I. L. , Diamond, A. W. , & Robertson, G. J. (2008). Annual survival of North American razorbills (*Alca torda*) varies with ocean climate indices. Canadian Journal of Zoology, 86, 51–61. 10.1139/Z07-113

[jane14227-bib-0043] Le, S. , Josse, J. , & Husson, F. (2008). FactoMineR: An R package for multivariate analysis. Journal of Statistical Software, 25, 1–18. 10.18637/jss.v025.i01

[jane14227-bib-0044] Louzao, M. , Gallagher, R. , Garcia‐Baron, I. , Chust, G. , Intxausti, I. , Albisu, J. , Brereton, T. , & Fontan, A. (2019). Threshold responses in bird mortality driven by extreme wind events. Ecological Indicators, 99, 183–192. 10.1016/j.ecolind.2018.12.030

[jane14227-bib-0045] Merne, O. J. (2002). Razorbill *Alca torda* . In C. Wernham , M. Toms , J. H. Marchant , J. Clark , G. Siriwardena , & S. R. Baillie (Eds.), The migration atlas: Movements of the birds of Britain and Ireland (pp. 401–404). T. & A.D. Poyser.

[jane14227-bib-0046] Morley, T. I. , Fayet, A. L. , Jessop, H. , Veron, P. , Veron, M. , Clark, J. , & Wood, M. J. (2016). The seabird wreck in the Bay of Biscay and southwest approaches in 2014: A review of reported mortality. Seabird, 29, 22–38. 10.61350/sbj.29.22

[jane14227-bib-0047] Newell, M. , Wanless, S. , Harris, M. P. , & Daunt, F. (2015). Effects of an extreme weather event on seabird breeding success at a North Sea colony. Marine Ecology Progress Series, 532, 257–268. 10.3354/meps11329

[jane14227-bib-0048] Newman, L. , Blockley, F. , Hewitt, J. , & Wood, M. (2021). Seabird monitoring on Skomer Island in 2021. The Wildlife Trust of South and West Wales and University of Gloucestershire.

[jane14227-bib-0049] Oro, D. (2014). Seabirds and climate: Knowledge, pitfalls, and opportunities. Frontiers in Ecology and Evolution, 2, 79. 10.3389/fevo.2014.00079

[jane14227-bib-0050] Owen, E. , Steinfurth, A. , & Hughes, R. (2023). Atlantic puffin *Fratercula Arctica* . In D. Burnell , A. J. Perkins , S. E. Newton , M. Bolton , T. D. Tierney , & T. E. Dunn (Eds.), Seabirds count: A census of breeding seabirds in Britain and Ireland (2015–2021) (pp. 409–425). Lynx Nature Books.

[jane14227-bib-0051] Parmesan, C. , & Yohe, G. (2003). A globally coherent fingerprint of climate change impacts across natural systems. Nature, 421, 37–42. 10.1038/nature01286 12511946

[jane14227-bib-0052] Pradel, R. (1993). Flexibility in survival analysis from recapture data: Handling trap‐dependence. In J.‐. D. Lebreton & P. M. North (Eds.), Marked individuals in the study of bird population (pp. 29–37). Birkhauser Verlag.

[jane14227-bib-0053] Priestley, M. D. K. , Ackerley, D. , Catto, J. L. , Hodges, K. I. , McDonald, R. E. , & Lee, R. W. (2020). An overview of the extratropical storm tracks in CMIP6 historical simulations. Journal of Climate, 33, 6315–6343. 10.1175/JCLI-D-19-0928.1

[jane14227-bib-0054] Priestley, M. D. K. , & Catto, J. L. (2022). Future changes in the extratropical storm tracks and cyclone intensiy, wind speed, and structure. Weather and Climate Dynamics, 3, 337–360. 10.5194/wcd-3-337-2022

[jane14227-bib-0055] Priestley, M. D. K. , Pinto, J. , Dacre, H. , & Shaffrey, L. C. (2017). The role of cyclone clustering during the stormy winter of 2013/14. Weather, 72, 187–192. 10.1002/wea.3025

[jane14227-bib-0056] Pritchard, R. , Hughes, J. , Spence, I. , Haycock, B. , & Brenchley, A. (2021). The birds of Wales. Liverpool University Press.

[jane14227-bib-0057] R Core Team . (2022). R: A language and environment for statistical computing. R Foundation for Statistical Computing. https://www.R‐project.org/

[jane14227-bib-0058] Reiertsen, T. K. , Layton‐Matthews, K. , Erikstad, K. E. , Hodges, K. , Ballesteros, M. , Anker‐Nilssen, T. , Barrett, R. T. , Benjaminsen, S. , Bogdanova, M. , Christensen‐Dalsgaard, S. , Daunt, F. , Dehnhard, N. , Harris, M. P. , Langset, M. , Lorentsen, S. H. , Newell, M. , Bråthen, V. S. , Støyle‐Bringsvor, I. , Systad, G. H. , & Wanless, S. (2021). Inter‐population synchrony in adult survival and effects of climate and extreme weather in non‐breeding areas of Atlantic puffins. Marine Ecology Progress Series, 676, 219–231. 10.3354/meps13809

[jane14227-bib-0059] Sandvik, H. , Erikstad, K. E. , Barrett, R. T. , & Yoccoz, N. G. (2005). The effect of climate on adult survival in five species of North Atlantic seabirds. Journal of Animal Ecology, 74, 817–831. 10.1111/j.1365-2656.2005.00981.x

[jane14227-bib-0060] Schreiber, E. A. , & Burger, J. (2002). Biology of marine birds. CRC Press.

[jane14227-bib-0061] Skalski, J. R. , Hoffmann, A. , & Smith, S. G. (1993). Testing significance of individual‐ and cohort‐level covariates in animal studies. In J.‐. D. Lebreton & P. M. North (Eds.), Marked indviduals in the study of bird population (pp. 9–28). Birkhauser Verlag.

[jane14227-bib-0062] St John Glew, K. , Wanless, S. , Harris, M. P. , Daunt, F. , Erikstad, K. E. , Strom, H. , & Trueman, C. N. (2018). Moult location and diet of auks in the North Sea inferred from coupled light‐based and isotope‐based geolocation. Marine Ecology Progress Series, 599, 239–251. 10.3354/meps12624

[jane14227-bib-0063] Sydeman, W. J. , Hunt, G. L. , Pikitch, E. K. , Parrish, J. K. , Piatt, J. F. , Boersma, P. D. , Kaufman, L. , Anderson, D. W. , Thompson, S. A. , & Sherley, R. B. (2022). African penguins and localized fisheries management: Response to Butterworth and Ross‐Gillespie. ICES Journal of Marine Science, 79, 1972–1978. 10.1093/icesjms/fsac116

[jane14227-bib-0064] Sydeman, W. J. , Poloczanska, E. , Reed, T. E. , & Thompson, S. A. (2015). Climate change and marine vertebrates. Science, 350, 772–777. 10.1126/science.aac9874 26564847

[jane14227-bib-0065] Sydeman, W. J. , Schoeman, D. S. , Thompson, S. A. , Hoover, B. A. , Garcia‐Reyes, M. , Daunt, F. , Agnew, P. , Anker‐Nilssen, T. , Barbraud, C. , Barrett, R. , Becker, P. H. , Bell, E. , Boersma, P. D. , Bouwhuis, S. , Cannell, B. , Crawford, R. J. M. , Dann, P. , Delord, K. , Elliott, G. , … Watanuki, Y. (2021). Hemispheric asymmetry in ocean change and the productivity of ecosystem sentinels. Science, 372, 980–983. 10.1126/science.abf1772 34045354

[jane14227-bib-0066] Votier, S. C. , Birkhead, T. R. , Oro, D. , Trinder, M. , Grantham, M. J. , Clark, J. A. , McCleery, R. H. , & Hatchwell, B. J. (2008). Recruitment and survival of immature seabirds in relation to oil spills and climate variability. The Journal of Animal Ecology, 77, 974–983. 10.1111/j.1365-2656.2008.01421.x 18624739

[jane14227-bib-0067] Votier, S. C. , Hatchwell, B. J. , Beckerman, A. , McCleery, R. H. , Hunter, F. M. , Pellatt, J. , Trinder, M. , & Birkhead, T. R. (2005). Oil pollution and climate have wide‐scale impacts on seabird demographics. Ecology Letters, 8, 1157–1164. 10.1111/j.1461-0248.2005.00818.x 21352439

[jane14227-bib-0068] Walz, M. , Befort, D. , Kirchner‐Bossi, N. , Ulbrich, U. , & Leckebusch, G. (2018). Modelling serial clustering and inter‐annual variability of European winter windstorms based on large‐scale drivers. International Journal of Climatology, 38, 3044–3057. 10.1002/joc.5481 31031527 PMC6473506

[jane14227-bib-0069] Wanless, S. , Albon, S. D. , Daunt, F. , Sarzo, B. , Newell, M. , Gunn, C. , Speakman, J. R. , & Harris, M. P. (2023). Increased parental effort fails to buffer the cascading effects of warmer seas on common guillemot demographic rates. Journal of Animal Ecology, 92, 1622–1638. 10.1111/1365-2656.13944 37212614

[jane14227-bib-0070] Wanless, S. , Harris, M. P. , Redman, P. , & Speakman, J. R. (2005). Low energy values of fish as a probable cause of a major seabird breeding failure in the North Sea. Marine Ecology Progress Series, 294, 1–8. 10.3354/meps294001

[jane14227-bib-0071] White, G. C. , & Burnham, K. P. (1999). Programme MARK: Survival estimation from populations of marked animals. Bird Study, 46, 120–139. 10.1080/00063659909477239

