# Supplementary material for: Long‐term multi‐species demographic studies reveal divergent negative impacts of winter storms on seabird survival
Source: J Anim Ecol. 2024 Nov 19;94(1):139–53. doi: 10.1111/1365-2656.14227 (PMC11729536; doi:10.1111/1365-2656.14227)
Supplement: Supplementary file 1 — Figure S1: Example immersion data plot used to determine timing of calibration periods. Figure S2: Example plot of raw light data from geolocators, which visualises timing of twilights recorded by GLS devices over time. Figure S3: Annual winter 90% UDs of all adult guillemots tracked. Figure S4: Annual winter 90% UD of all adult puffins tracked. Figure S5: Annual survival estimates and 95% profile likelihood confidence intervals for Atlantic puffins from the model ϕt pt/t. Figure S6: Annual resighting estimates of common guillemots, from the model ϕt p ./t. Figure S7: Annual resighting rate of razorbills, from the model ϕt p t/.. Figure S8: Annual resighting rate of Atlantic puffins, from the model ϕt p t + m. Table S1: Proportion of each annual 90% utilisation distribution (UD) that overlapped with the UD of another year. Table S2: Proportion of each annual 90% UD that overlapped with the UD of another year. Table S3: Results of PCA performed on storm variables extracted from each species' wintering area. Table S4: Storm variables significantly correlated with PC1 and the direction of the relationship. Table S5: Goodness of fit test results for guillemots, razorbills, and puffins. [file JANE-94-139-s001.docx]

**Supplement 1.** Full tracking data processing methods.

- 1. Guillemot data

Combined geolocation-immersion loggers (GLS) were deployed on guillemots breeding at the Amos between 2009 and 2013. A total of 31 migratory tracks that included the core wintering period from December – February were obtained from 22 individual birds. Raw data files downloaded from the GLS devices were decompressed using BAStrack software, and the resulting light files were processed in R statistical software (version 4.2.1, R Core Team, 2022), using the packages *TwGeos* (Lisovski, Wotherspoon and Sumner, 2016) and *FLightR* (Rakhimberdiev and Saveliev, 2022). Raw light files were processed in *TwGeos* to turn light data into timing of twilights (sunset/sunrise), using a light intensity threshold of 2.5 to separate day from night. Twilight files were then imported into *FLightR* to estimate geographic location from light data following the workflow outlined in Rakhimberdiev *et al.* (2017), and supplementary material of Lisovski *et al.* (2020).

Calibration periods were defined as the period birds were present at the colony, determined by inspection of the immersion data (Figure S1). The first calibration period lasted from the date of device deployment until the date of departure from the colony; and the second calibration period was from date of return to the colony until the onset of incubation. Birds were assumed to depart the colony when the daily proportion of saltwater immersion showed a marked and sustained increase (~July); and return to the colony when the daily proportion of saltwater immersion showed a sustained decrease (~March). The second calibration period was stopped at the onset of incubation to avoid any errors resulting from shading of the device. The start of incubation was identified by intermittent periods of shading in the light data (Figure S2). The model was constrained to the spatial extent 15^o^W – 3^o^E, and 43^o^N – 65^o^N, based on ring recoveries of Skomer guillemots, and winter distribution of guillemots from other UK colonies (Votier 2008, Buckingham *et al.*, 2022). Areas over 10km inland were excluded from the model as guillemots do not migrate or stage over land. The mean of migration distance was set as 750km (Harris & Swann 2002), and maximum distance allowed to move between twilights was limited to 51km (Erikstad *et al.*, 2018). The model was run with 1,000,000 particles.


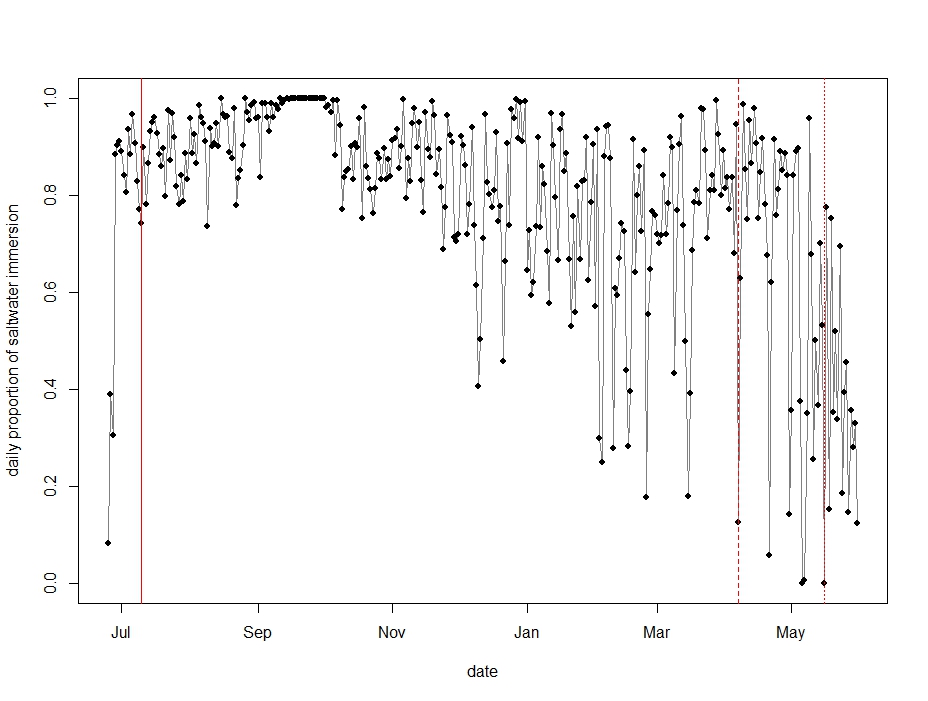


**Figure S1.** Example immersion data plot used to determine timing of calibration periods. The first calibration period was stopped on the date of the solid red line (first from left), as this is when there was a marked and prolonged increase in the daily proportion of time submerged, indicating that the bird had departed the colony. The second calibration period was started on the date of the dashed red line (second from left), as this is when the proportion of time wet decreases, suggesting the bird is attending the colony regularly. The second calibration was stopped on the date of the dotted red line (third from left), as this coincided with shading of the device on the light plot (Figure S2). Figures S1 and S2 have been taken from different birds – these plots were chosen as they were good, clear examples.


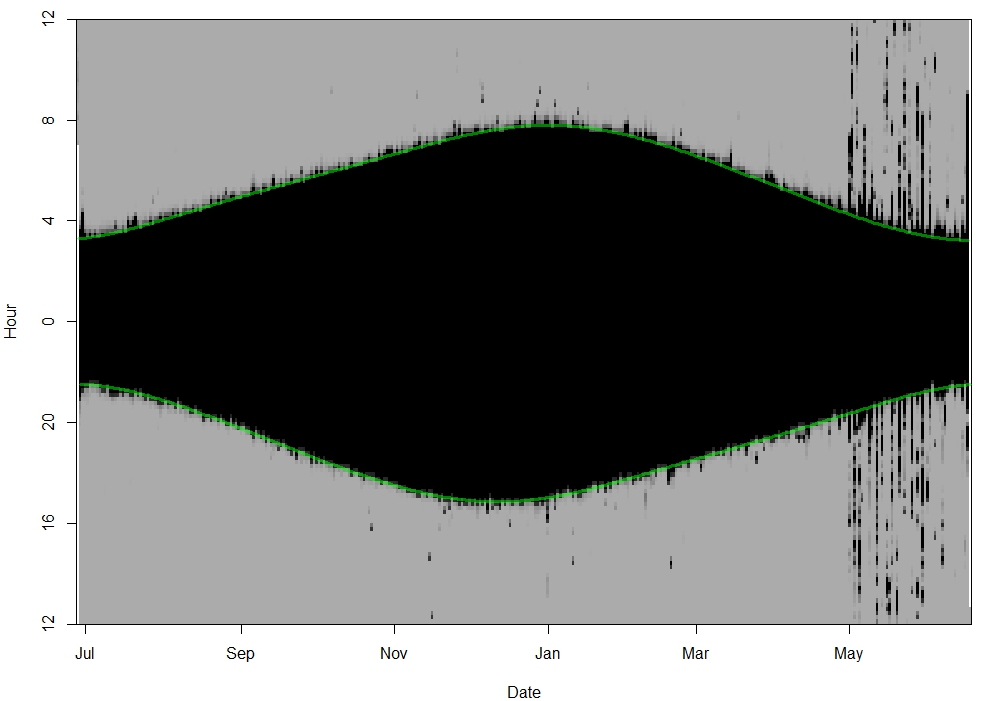


**Figure S2.** Example plot of raw light data from geolocators, which visualises timing of twilights recorded by GLS devices over time. Grey represents daylight, black represents night, so sunrises are along the top, sunsets along the bottom. The green line represents timing of twilights at Skomer, which closely follows timing of twilights experienced by the guillemot, indicating that the bird stayed close to Skomer throughout the non-breeding period. The plot also shows intermittent shading of the geolocator from May onwards, caused by the bird shading the device as it is incubating. Shading through daylight hours can cause issues with calibration, so the second calibration period (after the bird returned to Skomer) was stopped at the onset of shading in May.

- 1. Puffin Data

A total of 109 migratory tracks that included the December – February core winter period were obtained from GLS devices (models Mk13, Mk14, Mk18 [British Antarctic Survey], or Mk4083 [Biotrack]) deployed on 54 adult puffins breeding on Skomer between 2007 and 2014. Raw light data from the non-breeding season (August – March) were processed using BASTrack software and MatLab R2010b (Fayet et al. 2016). A speed filter of 500km per day (8 h of sustained flight at a mean speed of 64 km/h; Pennycuick 1997) was applied, and the 15 days either side of the autumn and spring equinoxes were removed (Fayet et al. 2016).

**Literature Cited**

Buckingham L, Bogdanova MI, Green JA, Dunn RE, Wanless S, Bennett S, Bevan RM, Call A et al. (2022) Interspecific variation in non-breeding aggregation: a multi-colony tracking study of two sympatric seabirds. Marine Ecology Progress Series*.* 684:181-197

Erikstad KE, Benjaminsen S, Reiertsen TK, Ballesteros M, Strom H (2018) *Modelling the movements of common guillemots and their chicks from Bjørnøya to the mainland coast of Norway*: Norwegian Institute for Nature Research.

Fayet AL, Freeman R, Shoji A, Boyle D, Kirk HL, Dean BJ, Perrins CM, Guilford T (2016) Drivers and fitness consequences of dispersive migration in a pelagic seabird. Behavioural Ecology*.* 27:1061-1072

Harris MP & Swann B (2002) Common Guillemot. In: Wernham C, Toms M, Marchant J, Clark J, Siriwardena G, Baillie S (eds) The migration atlas: movments of the birds of Britain and Ireland. London: T & AD Poyser. 397-400

Lisovski S, Wotherspoon S, Sumner M (2016) TwGeos: Basic data processing for light-level geolocation archival tags. R package version 0.1.2.

Lisovski S, Bauer S, Briedis M, Davidson SC, Dhanjal-Adams KL, Hallworth MT, Karagicheva J, Meier CM, et al. (2020) Light-level geolocation analyses: A user's guide. Journal of Animal Ecology. 89:221-236

Pennycuick C (1997) Actual and ‘optimum’ flight speeds: field data reassessed. Journal of Experimental Biology 200:2355-2361.

Rakhimberdiev E, Saveliev A, Piersma T, Karagicheva J (2017) FLightR: An R package for reconstructing animal paths from solar geolocation loggers. Methods in Ecology and Evolution. 8:1482-1487

Rakhimberdiev E, Saveliev A (2022) FLightR: SSM for solar geolocation. R package version 0.5.3.

R Core Team (2022) R: a language and environment for statistical computing. R Foundation for Statistical Computing, Vienna, Austria. URL <https://www.R-project.org/>

Votier SC, Birkhead TR, Oro D, Trinder M, Grantham MJ, Clark JA, McCleery RH, Hatchwell BJ (2008) Recruitment and survival of immature seabirds in relation to oil spills and climate variability. Journal of Animal Ecology 77:974-983.

**Supplement 2.** Estimating spatial overlap.

- 1. Guillemots

**Table S1.** Proportion of each annual 90% utilisation distribution (UD) that overlapped with the UD of another year.

|  | **2009** | **2010** | **2011** | **2012** |
| --- | --- | --- | --- | --- |
| **2009** | - | 0.833 | 1.000 | 0.750 |
| **2010** | 0.204 | - | 0.816 | 0.776 |
| **2011** | 0.069 | 0.229 | - | 0.280 |
| **2012** | 0.164 | 0.691 | 0.891 | - |


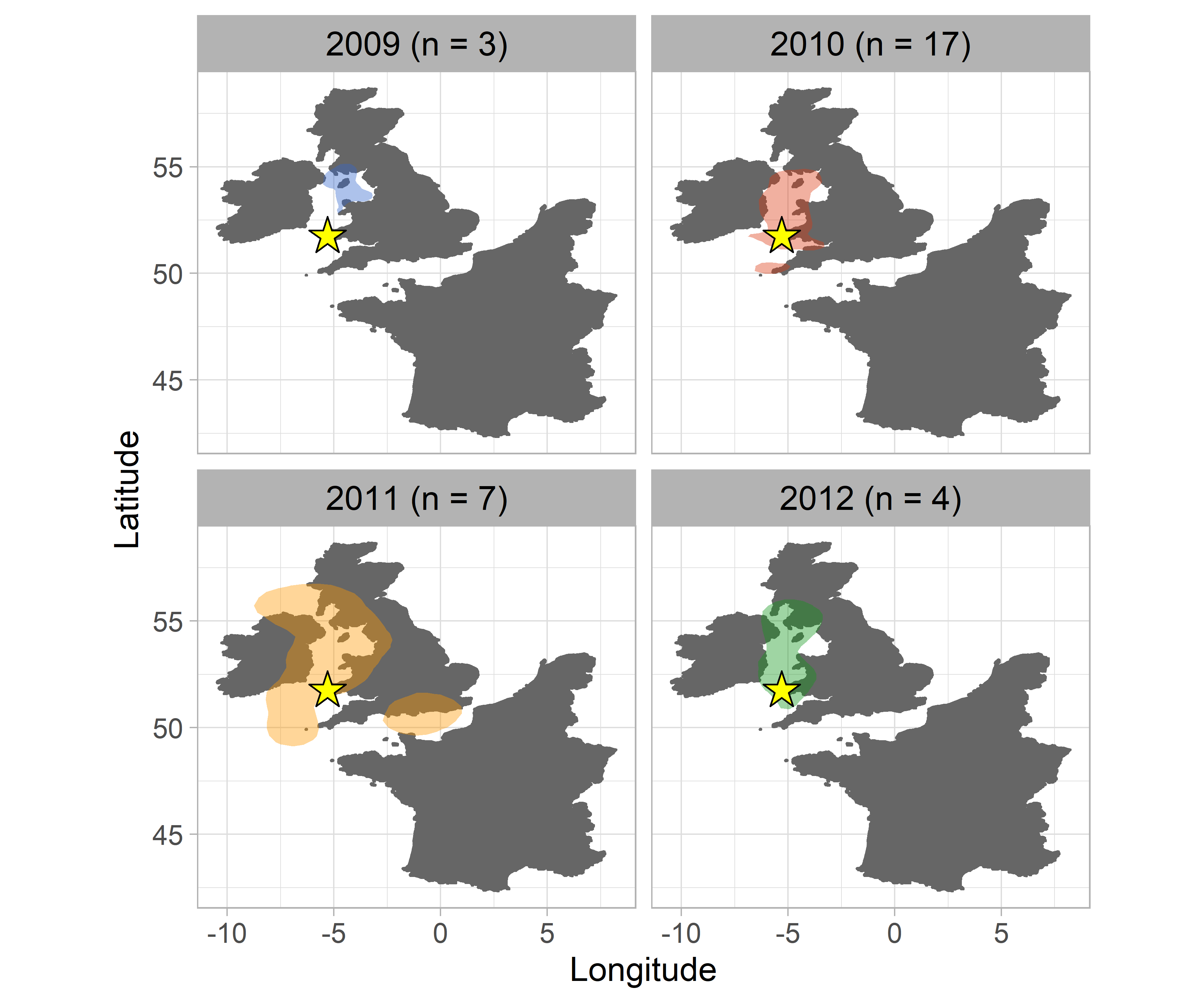


**Figure S3.** Annual winter 90% UDs of all adult guillemots tracked. The location of Skomer is indicated by the yellow star. As UDs showed a high degree of overlap, annual UDs were combined into one overall winter 90% UD.

- 1. Puffins

**Table S2.** Proportion of each annual 90% UD that overlapped with the UD of another year.

|  | **2007** | **2008** | **2009** | **2010** | **2011** | **2012** | **2013** |
| --- | --- | --- | --- | --- | --- | --- | --- |
| **2007** | - | 0.702 | 0.702 | 0.894 | 0.787 | 0.915 | 0.468 |
| **2008** | 0.550 | - | 0.800 | 0.867 | 0.850 | 0.933 | 0.683 |
| **2009** | 0.532 | 0.774 | - | 0.935 | 0.871 | 0.887 | 0.677 |
| **2010** | 0.483 | 0.598 | 0.667 | - | 0.793 | 0.851 | 0.540 |
| **2011** | 0.451 | 0.622 | 0.659 | 0.841 | - | 0.805 | 0.585 |
| **2012** | 0.500 | 0.651 | 0.640 | 0.860 | 0.767 | - | 0.616 |
| **2013** | 0.379 | 0.707 | 0.724 | 0.810 | 0.828 | 0.914 | - |


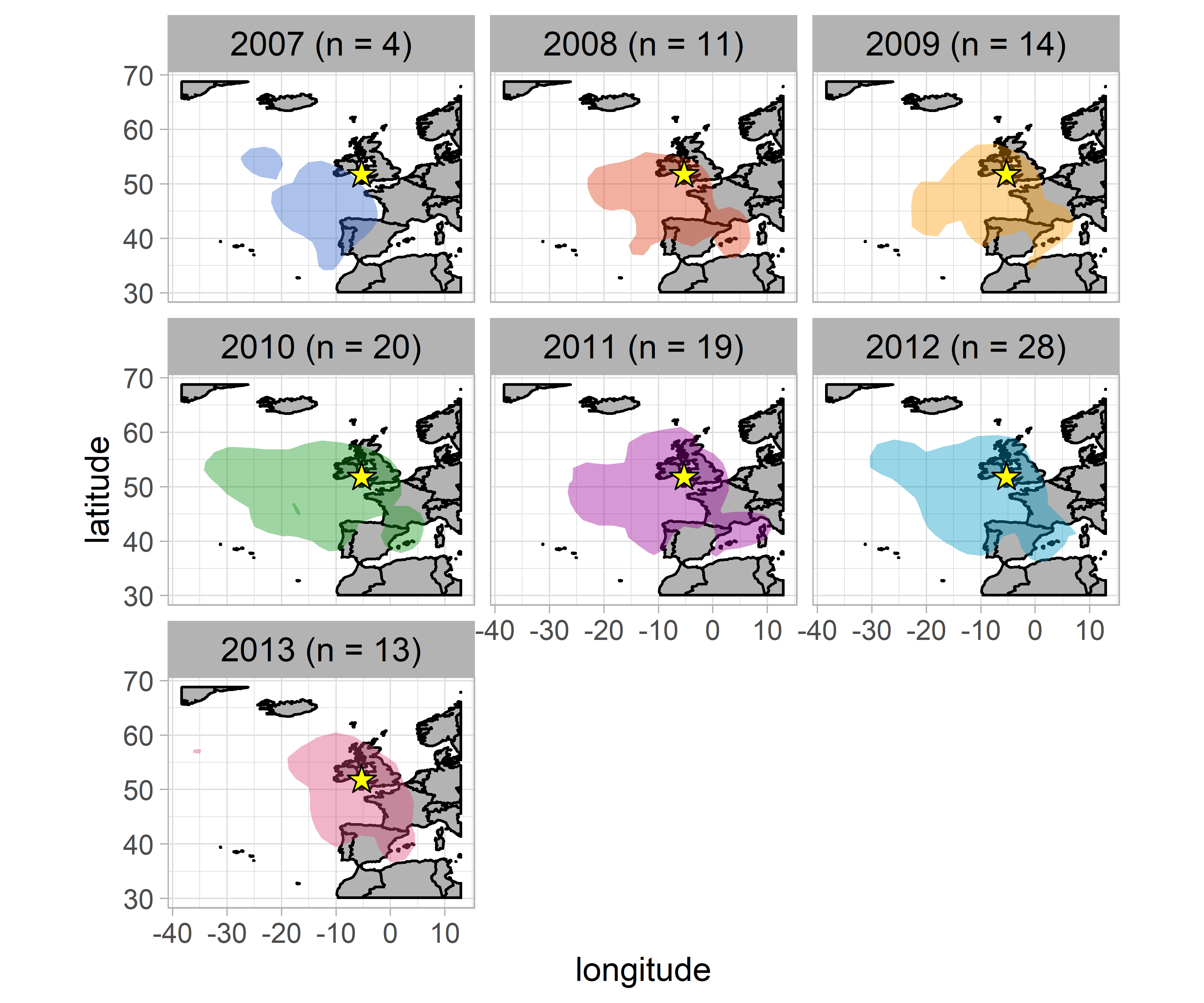


**Figure S4.** Annual winter 90% UD of all adult puffins tracked. The location of Skomer is indicated by the yellow star. As UDs showed a high degree of overlap, annual UDs were combined into one overall winter 90% UD.

**Supplement 3.** Principal Component Analysis (PCA) results.

**Table S3.** Results of PCA performed on storm variables extracted from each species’ wintering area.

|  | **Component** | **Eigenvalue** | **% Variance** | **Cumulative % Variance** |
| --- | --- | --- | --- | --- |
| **Guillemot** | PC1 | 2.957 | 49.282 | 49.282 |
|  | PC2 | 1.341 | 22.342 | 71.624 |
|  | PC3 | 0.760 | 12.662 | 84.286 |
|  | PC4 | 0.660 | 11.000 | 95.286 |
|  | PC5 | 0.191 | 3.188 | 98.474 |
|  | PC6 | 0.092 | 1.526 | 100 |
| **Razorbill** | PC1 | 2.734 | 45.561 | 45.561 |
|  | PC2 | 1.652 | 27.538 | 73.099 |
|  | PC3 | 0.858 | 14.301 | 87.400 |
|  | PC4 | 0.495 | 8.255 | 95.655 |
|  | PC5 | 0.167 | 2.782 | 98.436 |
|  | PC6 | 0.094 | 1.564 | 100 |
| **Puffin** | PC1 | 2.697 | 44.958 | 44.958 |
|  | PC2 | 1.819 | 30.324 | 75.282 |
|  | PC3 | 0.854 | 14.226 | 89.508 |
|  | PC4 | 0.453 | 7.551 | 97.059 |
|  | PC5 | 0.140 | 2.328 | 99.387 |
|  | PC6 | 0.037 | 0.613 | 100 |

**Table S4.** Storm variables significantly correlated with PC1 and the direction of the relationship.

| **Guillemots** | | **Razorbills** | | **Puffins** | |
| --- | --- | --- | --- | --- | --- |
| **variable** | **correlation** | **variable** | **correlation** | **variable** | **correlation** |
| total no. | 0.775 | days > 30m/s wind | 0.837 | days > 30m/s wind | 0.789 |
| days > 5m wave | 0.693 | total no. | 0.637 | days > 7m wave | 0.783 |
| days > 35m/s wind | 0.671 | days > 10m wave | 0.619 | total no. | 0.717 |
| mean duration | -0.531 | mean duration | -0.434 | mean gap | -0.628 |
| mean gap | -0.752 | mean gap | -0.658 | mean intensity | -0.732 |
| mean intensity | -0.761 | mean intensity | -0.788 |  |  |

**Supplement 4.** Goodness of fit results

**Table S5.** Goodness of fit test results for guillemots, razorbills, and puffins. Significant lack of fit (p < 0.05, ĉ > 3) is identified in bold. Test 3 tests for the presence of transience (marking of non-resident birds, or effects of marking on survival); and Test 2 tests for trap-dependence (heterogeneity in encounter probability between birds encountered or not at the previous occasion). ‘Global’ is the sum of all Test results. The trap effects model is the sum of all Test components excluding Test 2.CT.

|  |  | **Test 3.SR** | **Test 3.SM** | **Test 2.CT** | **Test 2.CL** | **Global** | **Trap effects model** |
| --- | --- | --- | --- | --- | --- | --- | --- |
| **Guillemot** | χ^2^ | 29.54 | 27.48 | 1431.80 | 88.97 | 1577.79 | 145.99 |
|  | df | 25 | 27 | 34 | 47 | 133 | 99 |
|  | ĉ | 1.182 | 1.018 | **42.112** | 1.893 | **11.863** | 1.475 |
|  | p-value | 0.242 | 0.287 | **<0.001** | **0.002** | **<0.001** | **0.002** |
| **Razorbill** | χ^2^ | 35.75 | 40.80 | 1081.21 | 144.43 | 1302.19 | 220.98 |
|  | df | 47 | 47 | 49 | 54 | 197 | 148 |
|  | ĉ | 0.761 | 0.868 | **22.066** | 2.675 | **6.610** | 1.493 |
|  | p-value | 0.885 | 0.726 | **<0.001** | **<0.001** | **<0.001** | **<0.001** |
| **Puffin** | χ^2^ | 80.366 | 89.021 | 2350.99 | 319.73 | 2840.10 | 489.12 |
|  | df | 48 | 53 | 47 | 111 | 259 | 212 |
|  | ĉ | 1.674 | 1.680 | **50.021** | 2.880 | **10.966** | 2.307 |
|  | p-value | **0.002** | **<0.001** | **<0.001** | **<0.001** | **<0.001** | **<0.001** |

**Supplement 5.** Puffin time-dependent survival estimates with full confidence intervals.


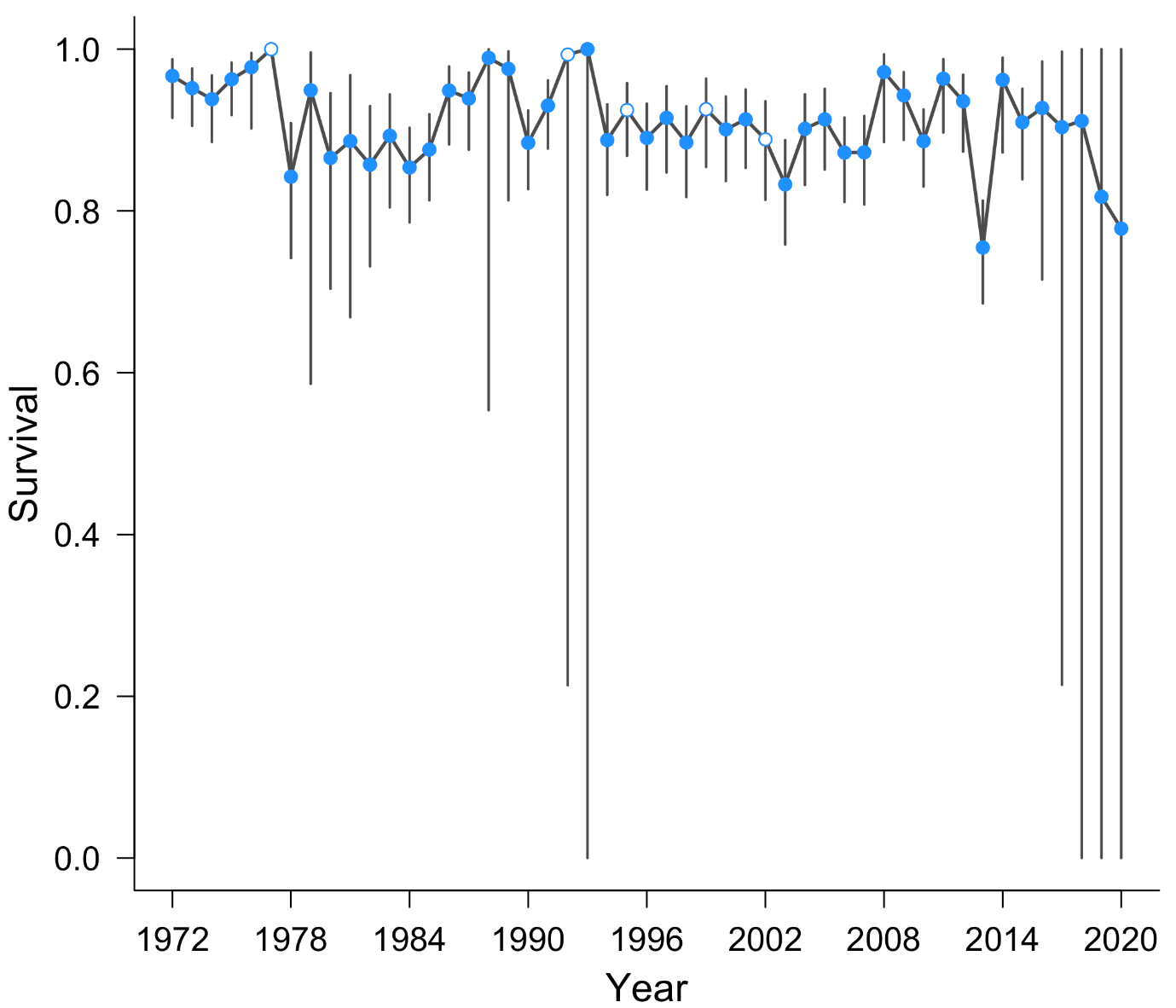


**Figure S5.** Annual survival estimates and 95% profile likelihood confidence intervals for Atlantic puffins from the model ϕ_t_ p _t/t_. Oil spill years are highlighted by the white points with blue outlines.

**Supplement 6.** Annual resighting effort.

**
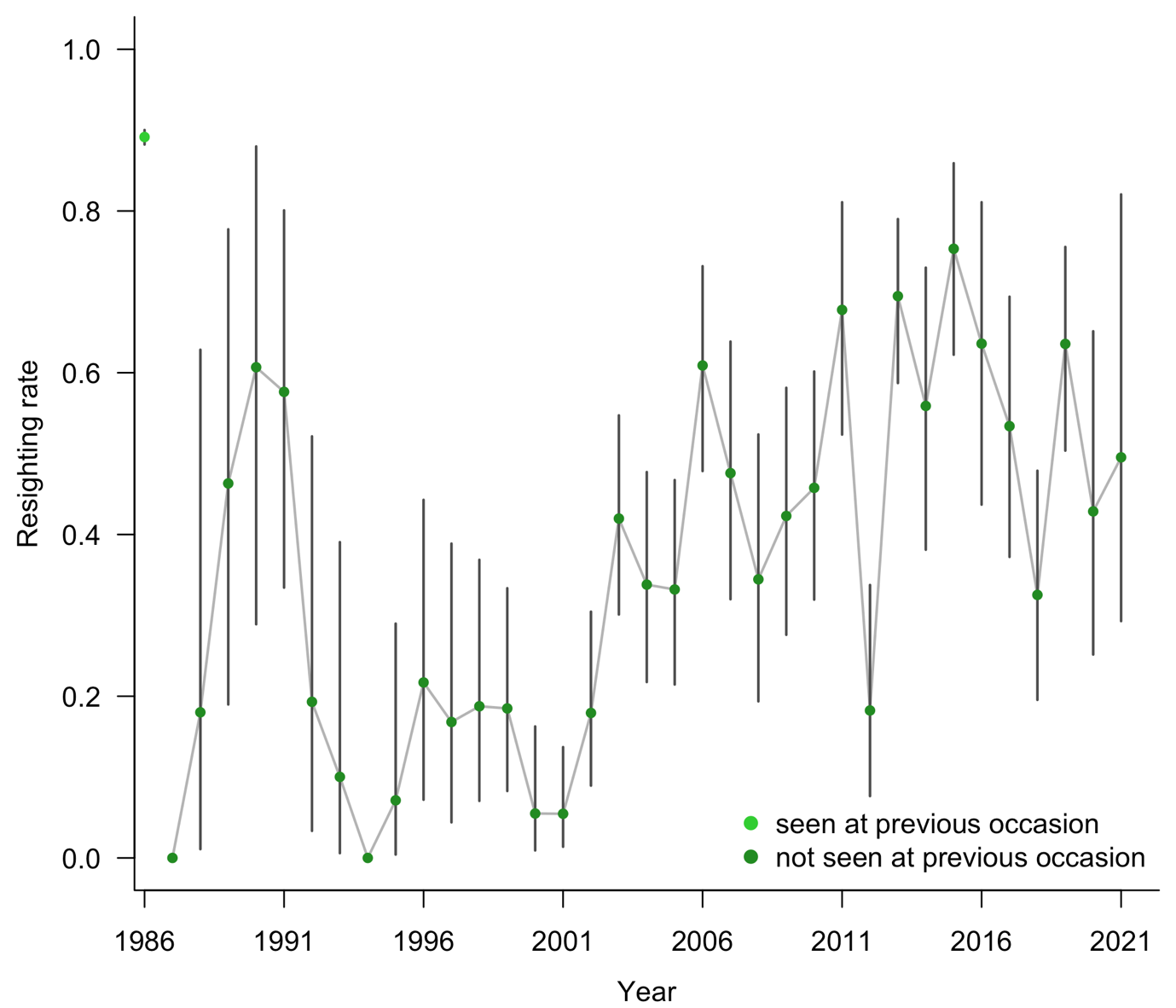
Figure S6.** Annual resighting estimates of common guillemots, from the model ϕ_t_ *p*_./t_ Resighting rate for birds seen at the previous occasion (before the forward slash) was held constant and is shown in light green. The dark green points represent the resighting rate for birds not seen on the previous occasion (after the forward slash), which varied over time. Error bars represent 95% profile likelihood confidence intervals.

**
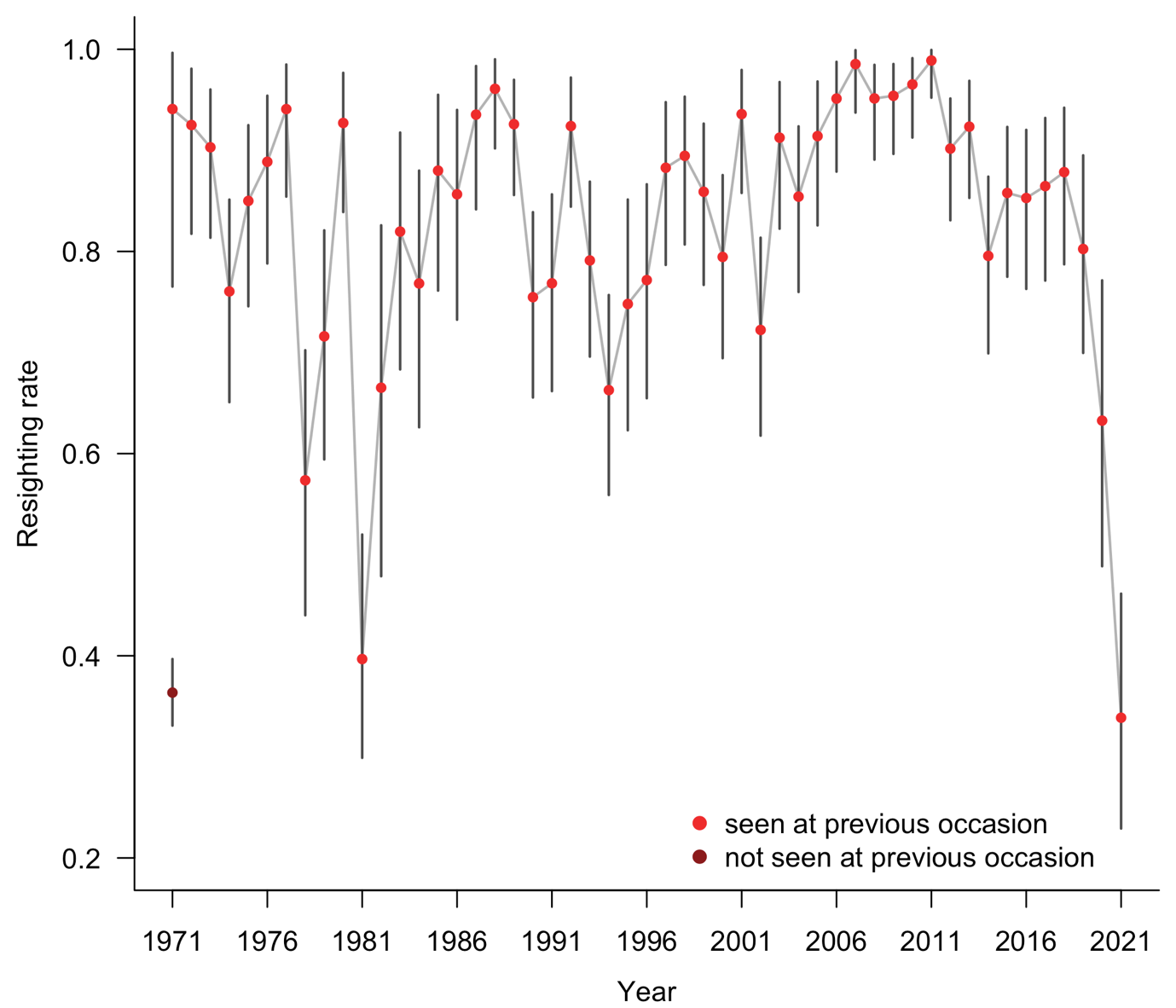
Figure S7.** Annual resighting rate of razorbills, from the model ϕ_t_ *p* _t/._. Resighting rate for birds seen at the previous occasion (before the forward slash) varied over time and is shown in light red. The dark red point represents the resighting rate for birds not seen on the previous occasion (after the forward slash), which was held constant. Error bars represent 95% profile likelihood confidence intervals.


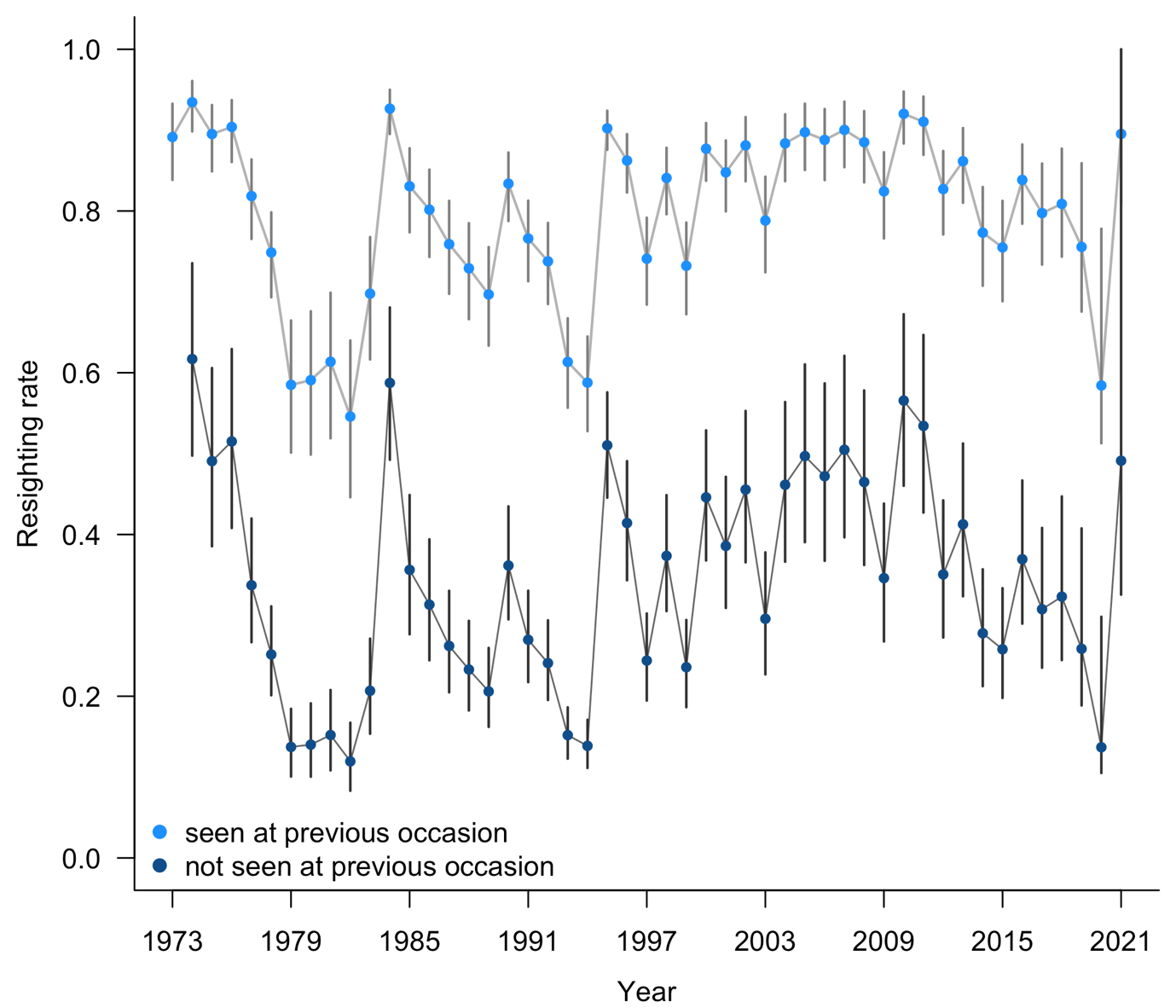


**Figure S8.** Annual resighting rate of Atlantic puffins, from the model ϕ_t_ *p* _t + m­_. Light blue points represent the resighting rate for birds seen at the previous sampling occasion, and dark blue points represent birds not seen at the previous occasion. Resighting rates varied over time, with an additive trap effect. Error bars represent 95% profile likelihood confidence intervals.

**Supplement 7 – interpreting m-arrays in supplementary data.**

The m-array summarises encounter histories into the number of individuals released on each occasion, and when and how many of them were resighted at subsequent occasions. The m-arrays for each species can be found in the supplementary data file, and here we explain how to interpret them using the m-array for guillemots as an example.

First, column R_i_ indicates the total number of individuals that were released at each sampling occasion. For example, at the first sampling occasion in 1985, 68 adult guillemots were ringed and released. Then, in 1986 a total of 69 individuals were released – this is comprised of 61 individuals from the first release in 1985 that were resighted in 1986, plus 8 newly ringed individuals (69-61 = 8). The m_i, j_ values are the number of individuals from a given release event which were resighted for the first time at a particular occasion. For example, m_1985, 1986_ = 61; or, 61 of the 68 individuals marked and released in 1985 were resighted for the first time in 1986. The values in column r_i_ are the total number of individuals resighted at least once from a given cohort. From the 1985 cohort, a total of 62 individuals were resighted over the study period. R_i_ – r_i­_ indicates the number of individuals that were never seen again, and the final column ri/Ri (%) expresses the proportion of individuals resighted again from each cohort as a percentage.
